# Supplementary material for: Crystal, Fivefold and Glass Formation in Clusters of Polymers Interacting with the Square Well Potential
Source: Polymers (Basel). 2020 May 13;12(5):1111. doi: 10.3390/polym12051111 (PMC7285265; doi:10.3390/polym12051111)
Supplement: Supplementary file 1 [file polymers-12-01111-s001.zip › paper_interactive_3D.pdf]

polymers

MDPI

Article

Crystal, Fivefold and Glass Formation in Clusters of Polymers Interacting with the Square Well Potential

Miguel Herranz, Manuel Santiago, Katerina Foteinopoulou, Nikos Ch. Karayiannis * and Manuel Laso

Institute for Optoelectronic Systems and Microtechnology (ISOM) and ETSI Industriales, Universidad Politécnica de Madrid (UPM), José Gutierrez Abascal 2, 28006, Madrid, Spain; miguel.herranzf@upm.es (M.H.); manuel.santiago.carrillo@alumnos.upm.es (M.S.); kfoteinopoulou@etsii.upm.es (K.F.); mlaso@etsii.upm.es (M.L.)

* Correspondence: nkarayiannis@etsii.upm.es; Tel.: +34-910677318

Received: 4 April 2020; Accepted: 6 May 2020; Published: date

Abstract: We present results, from Monte Carlo (MC) simulations, on polymer systems of freely jointed chains with spherical monomers interacting through the square well potential. Starting from athermal packings of chains of tangent hard spheres, we activate the square well potential under constant volume and temperature corresponding effectively to instantaneous quenching. We investigate how the intensity and range of pair-wise interactions affected the final morphologies by fixing polymer characteristics such as average chain length and tolerance in bond gaps. Due to attraction chains are brought closer together and they form clusters with distinct morphologies. A wide variety of structures is obtained as the model parameters are systematically varied: weak interactions lead to purely amorphous clusters followed by well-ordered ones. The latter include the whole spectrum of crystal morphologies: from virtually perfect hexagonal close packed (HCP) and face centered cubic (FCC) crystals, to random hexagonal close packed layers of single stacking direction of alternating HCP and FCC layers, to structures of mixed HCP/FCC character with multiple stacking directions and defects in the form of twins. Once critical values of interaction are met, fivefold-rich glassy clusters are formed. We discuss the similarities and differences between energy-driven crystal nucleation in thermal polymer systems as opposed to entropy-driven phase transition in athermal polymer packings. We further calculate the local density of each site, its dependence on the distance from the center of the cluster and its correlation with the crystallographic characteristics of the local environment. The short- and long-range conformations of chains are analyzed as a function of the established cluster morphologies.

Keywords: square well; polymers; molecular simulation; Monte Carlo; crystallization; cluster; close packing; fivefold; ordered phase; twinning; phase transition; face centered cubic; hexagonal close packed; hard sphere; glass

1. Introduction

The phase behavior of general atomic and particulate systems has a major influence on the macroscopic properties of the end materials. Thus, its understanding and eventual prediction are of paramount importance in technology and industry [1,2]. While being in the spotlight of human interest and research since early times, crystal nucleation and growth are still not fully understood, albeit being encountered in a plethora of distinct physical and chemical systems [3–7]. The primary factor behind this is that crystallization spans several length scales, starting from the clustering and ordering of atoms at the level of nano, to the macroscopic size of the fully formed crystal. Accordingly, its identification and successive quantification requires advanced experimental techniques. In parallel, theoretical models of crystal nucleation and growth have been developed to

predict how the processing conditions affect phase transition so as to identify the key parameters that dictate final morphologies [8,9]. Towards this direction, computer simulations [10,11] can play an important role: subject to the accuracy of the employed molecular model and the adequacy of the method/algorithm to provide sufficient observation time, very helpful insights can be gained on the connection between structure, at the level of atoms, and macroscopic phase behavior.

Crystallization of polymers is of special importance as chain crystals are formed under special processing conditions and confer unique properties to polymeric materials [12–15]. Apart from the obvious effect of chemical constitution of the repeat units, additional factors that affect polymer crystallization include chain length, molecular architecture (long- or short-chain branching), the presence of interfaces or nanoparticles and the processing conditions like the quench rate. In the last years, encouraged by the rise of polymer-based, thin-film materials, the effect of confined geometries on polymer crystallization has also gained a significant interest [16,17]. Despite the ever-growing body of published literature, there are still numerous and non-trivial open questions around this phenomenon, especially when complex macromolecular systems are involved. From the simulation perspective, the vast range of characteristic time and length scales encountered in bulk polymers adds a further difficulty in the modeling study. Apart from an exceptionally large collection of simulation works on chemically realistic macromolecular models, significant effort has been devoted to the modeling of chemically simpler, still physically relevant colloidal or granular polymers. In the simplest possible realization, polymers can be modeled as linear, freely-jointed chains of tangent hard spheres of uniform size [18–21].

During recent years, advances in synthesis and characterization of colloidal and granular polymers have renewed an interest in understanding the structure-property relationship in chemically simple macromolecular systems. Motivated by this, in the past we explored the salient features of entropically-driven, athermal polymer crystallization through extensive Monte Carlo (MC) simulations [22–27] and compared it with the phase behavior of monomeric counterparts [28,29]. Recently, we extended the modeling studies to hard-sphere polymers under confinement in at least one dimension [30–32].

In the present work we study the phase behavior of linear, freely jointed chains whose spherical monomers interact through the pair-wise, square well (SW) potential. Compared to hard spheres (HS) the SW model, apart from the excluded volume, includes attraction between atoms, or repulsion in the case of the square shoulder (SS) potential. Hence, the phase behavior of the corresponding system is expected to depend strongly on the intensity and range of such interactions. Furthermore, they lead to energy dominating the phase transition in thermal SW systems as opposed to entropy, which is the driving force in athermal HS counterparts. The SW model has been previously employed to study phenomena from clustering and self-assembly to crystallization. Regarding monomeric systems, Babu et al. [33] studied crystallization of monomeric spheres with SW potential through Brownian cluster dynamics. Klotsa and Jack [34] investigated self-assembly of SW monomers into crystals to predict phase behavior. Through Molecular Dynamics (MD), Takada and Hayakawa [35] showed the coalescence of particles in clusters by the application of SW potential under shear flow. Sevick and Monson [36] studied cluster formation of square well particles through theoretical considerations. Models that are more complex can be used, such as the flat-well potential used for 2D structural analysis [37–39], or the double-Gaussian potential applied in the characterization of structural collapse of monomers [40]. The sticky model is a specific case of the square well potential obtained by collapsing the attraction range, and has been investigated extensively in past studies [41–45].

Beyond monomeric systems, the SW potential/Janus dumbbell has also been applied to study self-assembly of dimers [46,47]. With respect to chain systems, Kuriata and Sikorski [48] adopted the SW potential to study the collapse transition of cyclic homopolymers and block copolymers, whereas Zierenber et al. [49] investigated aggregation of dilute semi flexible chains driven by temperature. Regarding single chains, previous works explored the thermodynamics and phase transition of single homopolymers under the SW potential [50–53]. Schnabel and Bachmann have presented results on the conformation of perfect icosahedral conformations for specific chain lengths [54–56], as well as

on the effect of the interaction range of SW potential in chain systems [57]. Such structural analysis dates back in the early works in [58–60] on how the interaction type and range govern structural features of model atomic systems. Prabhu et al. [61] applied a patchy SW model in the study of step-growth polymerization. Results obtained in the present work can be further compared against the ones, obtained through MD simulations, on the competition between crystal and glass formation as a function of quench rate in bulk systems of bead-spring polymer chains [62]. The model of [62] serves effectively as a bridge between discontinuous models (SW and HS), as the ones presented here, and more realistic representations (like the Lennard-Jones (LJ)) as it incorporates tunable chain flexibility [63], commensurability between the average bond length and the monomer size and a truncated and shifted LJ potential for non-bonded interactions.

The present work focuses on the identification and characterization, through proper structural metrics, of thermodynamically stable morphologies, generated in extensive Monte Carlo simulations, as we tune the intensity and range of the attractive SW interaction between monomers. The observed polymer clusters vary from completely amorphous systems, and fivefold-rich glasses all the way to perfect crystals in a striking similarity not only to dense hard sphere counterparts [22,23,25,27,29] but also to glass and crystal formation encountered in chemically more realistic polymers systems under quenching at various rates [62].

The present manuscript is organized as follows: Section 2 details the model used, the simulated systems, the MC suite employed, and the metric used to characterize local structure. Section 3 reports the results on the phase behavior of the equilibrated, computer-generated system configurations and on the corresponding thermodynamically stable morphologies. Finally, Section 4 summarizes the main conclusions of the present work and discusses briefly current efforts and future plans.

2. Molecular Model, Simulation Algorithm and Systems Studied

In the present work we adopt the freely jointed model of linear chains of spherical monomers of uniform collision diameter σ_1, which also serves as the characteristic length of the system. Bond lengths, l, can fluctuate uniformly in the interval $l \in [\sigma_1, \sigma_1 + dl]$, where dl is the gap between successive spheres along the chain backbone. In the case of $dl \to 0$ the tangent limit is reached, corresponding numerically to a value of 10^{-5}. It has been found in previous works [27], [64] that allowing for bond gaps, as opposed to strict tangency, has a strong effect on the ability of athermal chains to crystallize. Here, a fixed value of $dl = 6.5 \times 10^{-4}$ is considered for all systems studied. Given the freely jointed model there is no potential governing bending or torsion angles. All pairs, including the bonded ones, interact with the square well (SW) / shoulder (SS) pair-wise potential [65] of the form:

$$v(r_{ij}) = \begin{cases} 0 & r_{ij} \geq \sigma_2 \\ -\varepsilon & \sigma_1 \leq r_{ij} < \sigma_2 \\ \infty & r_{ij} < \sigma_1 \end{cases} \quad (1)$$

where r_{ij} is the distance between the centers of spheres i and j and $v(r_{ij})$ is the energy. Parameters σ_1 and σ_2 in Equation (1) correspond to the collision distance and interaction range, respectively, while ε is the interaction intensity (depth of the well (SW) or height of the shoulder (SS)). Negative values of ε correspond to repulsion (SS) and positive ones to attraction (SW). By setting $\varepsilon = 0$ (and $\sigma_1 = \sigma_2$ for computational expedience) the SW/SS potential is reduced to the HS one. The limit of vanishing attraction range (setting $\sigma_2 \to \sigma_1$) results in sticky interactions. Although the SW/SS model is highly idealized and lacks chemical information, it still allows study of the effect of short-range interactions on the ability of chains to crystallize. Due to its simplicity, it can be causally linked to the athermal HS model, effectively allowing for a direct comparison between entropy- and energy-driven crystallization.

From the algorithmic perspective all simulations are based on a Monte Carlo (MC) suite [32] built around chain-connectivity-altering moves (CCAMs) [66,67] and a wall-wrapping algorithm. Very recently, it allowed the generation and equilibration of athermal polymer thin-films under conditions of extreme confinement [31,32]. Here, we employ a newer and extended version which can be employed in the simulation of polymer nanocomposites in the bulk and at interfaces. Two key

features of the latest code implementation, which are relevant to the present work, include: 1) the straightforward application of any type of isotropic, pair-wise potential, 2) the inclusion of cluster-based MC moves. The localized moves and CCAMs at the core of the MC scheme have been presented in detail in [32,68] and their efficiency is well documented under a variety of simulation conditions even for very long and/or definitely entangled chains (N = 1000) near the maximally random jammed (MRJ) state and/or under extreme confinement.

Two cluster moves are added in the current MC scheme: i) cluster displacement and ii) cluster rotation. For both moves to function, in the first step cluster detection is employed in the general case according to criteria of proximity and similarity. In the present work only proximity criteria are considered with a threshold distance being set at 1.2. The cluster detection algorithm proceeds iteratively by adding monomers in the clusters and merging them, when applicable, until no further additions are possible. Out of all possible clusters, one is selected randomly, and it is either randomly rotated or displaced. In the case that all chains form a single cluster the corresponding moves are automatically deactivated. At regular intervals (10^7 steps) cluster identification recurs; whenever more than one cluster is detected, cluster moves are activated again, and the cycle is repeated.

For all systems, the MC moves and their attempt probabilities are as follows: (i) rotation (10%), (ii) reptation (10%), (iii) flip (34.7%), (iv) intermolecular reptation (25%), (v) configurational bias (20%), (vi) simplified end-bridging (0.1%), (vii) simplified intermolecular end-bridging (0.1%) and cluster moves (0.1%). All local MC moves are executed in a configurational bias pattern as in Refs. [32] and [68] with the number of trial configurations per attempt being equal to $n_{dis} = 5$.

Initial configurations correspond to fully equilibrated random packings of hard-sphere chains at dilute conditions. Packing density, ϕ, is defined as the volume occupied by all monomers divided by the total volume of the simulation cell. In all simulations to be reported below, density, and thus system volume, is kept constant at a value of ϕ = 0.05. The average chain size is N = 12 with chain lengths allowed to fluctuate with equal probability in the interval $N \in [6,18]$. Cubic simulation cells are filled with 100 chains for a total of 1200 interacting monomers. Once the SW potential is activated the system becomes thermal and temperature, T, should be considered. For simplicity, all simulations conducted here are carried out at constant temperature ($T = 1 / k_B$, where k_B is the Boltzmann constant). Changing the initial athermal system to one at finite temperature corresponds to instantaneous quenching, with the intensity of the force field playing the role of an effective rate: the higher the ε value the higher the temperature difference; in other words, the lower the effective temperature reached by the quenching. This allows the comparison of the present results not only with the dense hard-sphere chain packings of athermal nature but further with more realistic thermal bead-spring LJ chain systems whose phase behavior is strongly dependent on quench rate [62,63,69].

Technical details on the simulation algorithm and a thorough exploration of the effect of physical parameters not considered here (chain length, density, temperature, bond gaps, confinement and nanofillers) on the short- and long-range structure of SW chains and on their phase behavior will be presented in the future.

For a fixed range ($\sigma_2 = 1.2$) we vary the intensity, ε, from 0.0 to 10.0; for a fixed intensity (ε = 0.5) we vary the range, σ_2, in the interval from 1.1 to 6.0. The limiting case of $\varepsilon = 0$ ($\sigma_2 = \sigma_1$) corresponds to hard spheres. The full parameter set, as explored in the present work, is summarized in Table 1.

Table 1. Pairs of interaction intensity, ε, and range, σ_2, as studied in the present work for the 100-chain $N = 12$ system at $\phi = 0.05$ and $k_B T = 1$.

				ε					
$\sigma_2 = 1.2$	0.0	1×10^{-5}	1×10^{-4}	1×10^{-3}	1×10^{-2}	0.1	0.2	0.3	0.4
	0.5	0.6	0.7	0.8	0.9	1.0	1.1	1.2	1.3
	1.4	1.5	1.6	1.8	2.0	4.0	6.0	8.0	10.0
				σ_2					
$\varepsilon = 0.5$	1.1	1.2	1.3	1.4	1.6	1.8	1.9	2.0	2.3
	2.4	2.6	2.7	2.8	2.9	3.0	3.2	3.5	4.0
	5.0	6.0							

System configurations, including energy and thermodynamic data are recorded every 10^7 steps and the corresponding simulation trajectories consist, on average, of 12×10^4 frames reaching total times in the order of 10^{12} steps. In a post-processing step recorded configurations (frames) are analyzed to quantify the degree of crystallinity and to identify the resulting structures. Towards this, we employ the Characteristic Crystallographic Element (CCE) norm [70] to gauge local structure. The CCE-based descriptor quantifies both orientational and radial deviations in the arrangement of atoms (or particles) with respect to a specific crystal structure. In a first step, the CCE norm utilizes the Voronoi cell (polyhedron) around each site to identify the closest neighbors. Local density, to be reported below, can be calculated as the reciprocal of the volume of the Voronoi polyhedron. In a second step, geometric symmetry operations are performed on each site and its neighbors, in order to identify a distinguishing set (fingerprint) of crystallographic elements (mirror planes, rotation and inversion axes, etc.) characteristic of each crystal type [71]. Given the expected formation of close packed structures, we have employed the CCE norm to identify similarity with respect to the HCP, and FCC crystals and to the non-crystallographic fivefold (FIV) local structure. The corresponding crystallographic elements and operations along with the algorithmic implementation of the CCE norm can be found in Refs. [23,28,29,70].

Once the CCE descriptor is applied, a number (norm), μ, is obtained for each site j, with respect to a specific crystal X. The closer μ_j^X is to zero, the greater the similarity to this specific crystal. As in the past works on non-overlapping spheres, we set a threshold value of $\mu^c \leq 0.245$ below which the site is considered of X-type similarity (X being HCP, FCC or FIV). Due to the discriminating nature of the CCE metric, no site can have simultaneously low CCE norm values with respect to two different reference crystals (i.e., if $\mu_j^X < \mu^c$ then $\mu_j^Y > \mu^c, Y \neq X$). Sampled over all sites in the system, we can further assign the corresponding probability distribution function, $P(\mu^X)$ for each recorded frame. The degree of crystallinity, τ^{cryst} can be considered as the sum of the order parameters, S^X, for the HCP and FCC crystals according to the following equation:

$$\tau^{\text{cryst}} = S^{\text{HCP}} + S^{\text{FCC}} = \int_0^{\mu^c} P(\mu^{\text{HCP}}) d\mu^{\text{HCP}} + \int_0^{\mu^c} P(\mu^{\text{FCC}}) d\mu^{\text{FCC}} \quad (2)$$

Phase transitions can be detected by observing the evolution of the degree of crystallinity (Equation (2)) as a function of MC frames; more importantly, we can label each and every site of the system as crystal (HCP or FCC), fivefold (FIV) or amorphous (neither HCP/FCC nor FIV). Visualization of the corresponding system configurations allows to further identify, with high precision, either crystal type and/or amorphous and fivefold-abundant, arrested (glassy) structures and the corresponding morphologies.

An important difference with respect to athermal packings at high density [22–24,27] and quenched bulk LJ chain systems [62] is that the system studied here is highly heterogeneous and monomers that lie on the external surface of the cluster possess incomplete neighbor environments, i.e., a coordination number lower than in a crystal, so that the CCE norm attains unrealistic high values. This affects the calculation of the degree of crystallinity as the ratio of the close packed sites by the total population (Equation (2)). As an alternative one could consider only spheres with

complete Voronoi polyhedron, excluding the ones on the external surface of the clusters. However, as we are interested in the phase transition, the exact value of τ^{cryst} is not as important as its qualitative evolution compared to the initial state. Accordingly, in the CCE-related calculations of τ^{cryst}, to be presented below, all spheres, including the ones on the cluster surface, are considered.

3. Results

3.1. Computer-Generated System Configurations

As mentioned above, initial configurations are generated as configurations of chains of tangent hard spheres in the bulk: 100-chain $N = 12$ at $\phi = 0.05$ with molecular lengths fluctuating uniformly in the interval $N \in [6,18]$. Given this structure, we activate the square well potential and vary the intensity and range of interactions as shown in Table 1 leading to instantaneous quenching. Due to the monomer attraction chains adopt coiled configurations and are brought closer together. Clusters containing monomers of different chains start to form. Given that the bond length (= 1) is smaller than the distance threshold for cluster detection (= 1.2) the minimum size of cluster cannot be lower than the shortest possible chain length (= 6) nor can the maximum number of clusters be higher than the number of chains (= 100). Depending on the values of ε and σ_2 clusters may become stable and further aggregate into larger groups. As the cluster size increases the corresponding cluster population drops. At sufficiently high intensities this aggregation eventually leads to the formation of a single cluster consisting of all chains in the system. An important difference with respect to single-chain simulations is that here, as we start from a bulk chain system, cluster growth occurs through merging of polymer chains rather than by shell-by-shell growth accompanied by compaction of the inner core. From the technical perspective the inclusion of cluster-based moves (displacement and rotation) in the MC protocol guarantees that the cluster unification occurs within modest computational time. Typical snapshots of computer-generated SW chain configurations as they evolve with simulation time (in MC steps) are shown in Figures 1 and 2. In the panels of the former figure sphere centers are subjected to periodic boundary conditions while in the latter they are unwrapped in order to visualize the shape and size of the cluster assemblies.

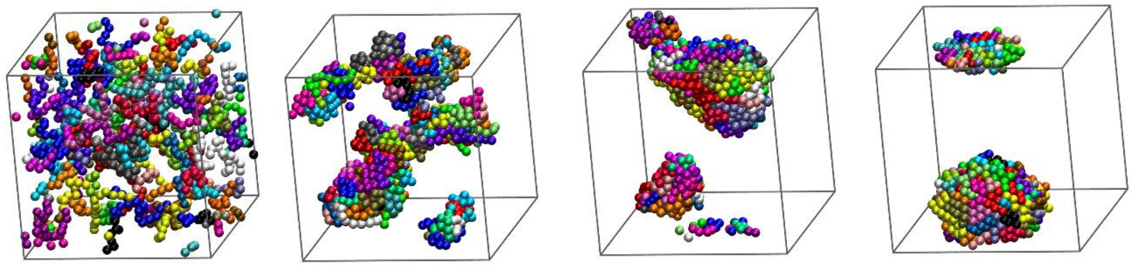

(**a**) (**b**) (**c**) (**d**)

Figure 1. (**a**) Snapshot of the athermal polymer system (100-chain $N = 12$, $\phi = 0.05$) serving as the initial configuration for all successive Monte Carlo simulations on chains interacting with the square-well (SW) potential. (**b**–**d**) System configurations for the SW chain system ($\varepsilon = 1.0$ and $\sigma_2 = 1.2$) after 10^6, 2×10^8 and 1.2×10^{12} Monte Carlo moves, respectively. The number of clusters is (**a**): 73, (**b**): 8, (**c**): 3 and (**d**): 1. The formation of a stable single cluster occurs at approximately 5×10^9 steps. Monomers are color-coded according to their parent chain. Coordinates of sphere centers are subjected to periodic boundary conditions in all dimensions. Image created with the VMD software (version 1.9.3, Theoretical and Computational Biophysics Group, University of Illinois, Urbana, IL, USA) [72]. 3-D, interactive versions of the figure panels exist in supplement material (as well as a 3-D, interactive version of the present manuscript).

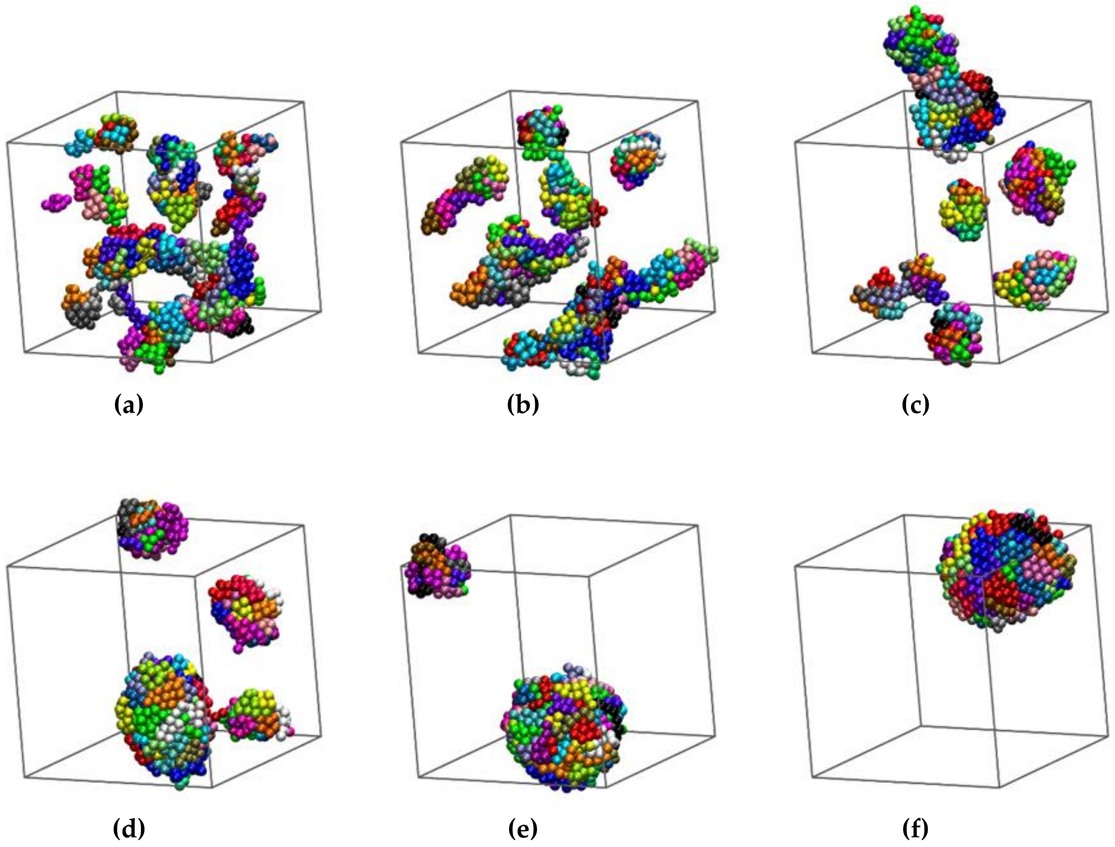

Figure 2. Snapshots of system configurations as simulation evolves for chains interacting with the square well potential (ε = 1.0 and σ_2 = 1.2) at (**a**): 10^5 [16], (**b**) 10^6 [8], (**c**) 10^7 [7], (**d**) 10^8 [4], (**e**) 10^9 [2] and (**f**) 1.4 × 10^{12} (end of simulation) [1] MC steps. Numbers in square brackets denote the number of distinct clusters. Monomers are color-coded according to their parent chain. Spheres of each cluster are shown in unwrapped coordinates. 3-D, interactive versions of the figure panels exist in supplement material (as well as a 3-D, interactive version of the present manuscript).

For weak attraction and/or short-range of interactions, individual clusters are small and may lie far from each other. In addition, when they meet their connection points (bridges), usually in the form of a single monomer, they can be easily deleted in successive steps due to the reversibility of the MC scheme. Clusters of initial random shape and size grow either by appending individual chains or through merging with other clusters. Again, depending strongly on the applied interaction parameters, the final single cluster may become more spherical and more symmetric compared to clusters at intermediate stages. Visual inspection of the final single cluster, as seen wrapped in panel (d) of Figure 1 or unwrapped in panel (f) of Figure 2, reveals further the formation of well-ordered patterns. Such ordering is, in general, absent in clusters of small size.

3.2. Crystallization

To detect any possible phase transition we employ the CCE metric to gauge the local environment around each monomer, *i*, by calculating the corresponding HCP, FCC and FIV norms: μ_i^{HCP}, μ_i^{FCC} and μ_i^{FIV}. Then, the corresponding distribution functions, $P(\mu_i^{\mathrm{HCP}})$, $P(\mu_i^{\mathrm{FCC}})$ and $P(\mu_i^{\mathrm{FIV}})$ can be obtained by sampling over all spheres for each frame. Finally, by imposing the threshold value (μ^c = 0.245) we can calculate the corresponding order parameters S^{HCP}, S^{FCC} and S^{FIV} and the degree of crystallinity, τ^c (Equation (2)).

Figure 3 shows the CCE probability densities for the last configuration for four different SW chain systems: (a) ε = 0.6 and σ_2 = 1.2, (b) ε = 1.0 and σ_2 = 2.0, (c) ε = 2.0 and σ_2 = 1.2 and (d) ε = 0.5 and σ_2 = 2.0. All corresponding simulations start from the same initial configuration, that of hard-sphere chains as seen in panel (a) of Figure 1, but the final structures, as revealed by the CCE analysis, are

quite different. Starting from the system (ε = 0.6 and σ_2 = 1.2) according to panel (a) of Figure 3 no site shows a CCE norm lower than the threshold value. Hence, there is no local environment with HCP-, FCC- or FIV- similarity. Based on this we can conclude that the assembled clusters have not crystallized. For comparison in the (ε = 1.0 and σ_2 = 1.2) system (panel (b) of Figure 3) an appreciable fraction of sites has low HCP- and FCC- norms. This is a clear sign of crystallization. The amounts of HCP and FCC are quite similar with a small preference toward HCP. Fivefold sites also exist in smaller, but non-negligible, quantities. Based on the above, and before even performing a visual inspection of the structures, we expect random stacking of compactly packed layers of monomers. The final ordered morphology for (ε = 1.0 and σ_2 = 1.2) should be faulted twin sectors of randomly alternating HCP or FCC character. This conclusion, to be supported in the continuation, is based on past findings that the presence of fivefolds is incompatible with extensive rhcp layering having a single stacking direction [26,28,29].

By keeping constant the interaction range and by further increasing the intensity (ε = 2.0 and σ_2 = 1.2, as shown in panel (c) of Figure 3), a different behavior is exhibited: fivefold sites are now abundant exceeding the populations of HCP- and FCC-like sites. In fact, their presence and stability hinder further crystal growth. This situation does not change even if the MC simulations are prolonged to simulation times (steps) two or three times longer than the ones to be reported below. The finding that the short-range, fivefold order competes against crystallization further verifies past simulation-based conclusions that fivefold symmetry can inhibit crystal nucleation and growth especially when such sites are abundant and/or they self-assemble into organized structures in hard-sphere [28,29] and LJ [62] chain systems. For sufficiently high attraction intensity the infinitely fast quench, which takes place at the start of the simulation, leads the chain cluster system to vitrification, instead of crystallization. This is in excellent qualitative agreement with the established MD results of Ref. [62]. In panel (d) of Figure 3 the system (ε = 2.0 and σ_2 =1.2) is displayed. The final configuration shows a remarkably high fraction of sites having an HCP character with no analogous sites of FCC or FIV similarity. Hence, this combination of interaction parameters leads clearly to crystallization with the final state being a perfect HCP crystal. As will be demonstrated later, similarly perfect FCC morphologies are also formed for specific pairs of intensity and range of interactions. This is not surprising given the minute difference in the thermodynamic stability of the two crystals.

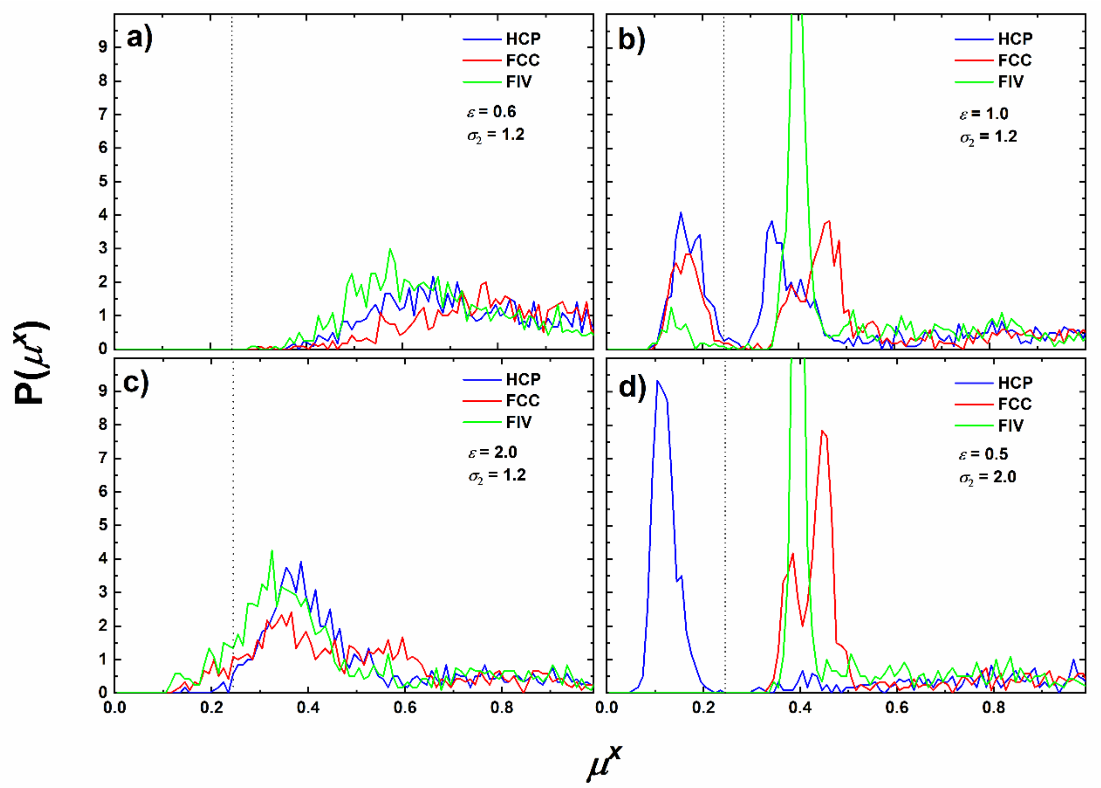

Figure 3. Probability distribution of the HCP- (blue), FCC- (red) and FIV- (green) CCE norm as obtained from structural analysis on the final system configuration for the SW chain systems: (**a**) ε = 0.6 and σ_2 = 1.2; (**b**) ε = 1.0 and σ_2 = 1.2; (**c**) ε = 2.0 and σ_2 = 1.2; (**d**) ε = 0.5 and σ_2 = 2.0. The vertical dotted line denotes the threshold value (μ^c = 0.245) for the identification of HCP, FCC or FIV local environments of non-overlapping spheres.

The evolution of the CCE order parameters, S^X, (X being HCP, FCC or FIV) as a function of MC steps can be seen in Figure 4 for the same representative systems as in Figure 3 except for (ε = 0.6 and σ2 = 1.2). The latter is not shown because the corresponding order parameters do not vary with simulation time, as the system remains amorphous with no traces of crystalline or fivefold domains; thus, the S^x-*versus*-MC steps curves practically coincide with the x-axis.

We should again note that according to Equation (2) by adding up the HCP and FCC order parameters we can estimate the degree of crystallinity, τ^c. Hence, by monitoring the evolution of these curves as simulation advances, we can identify the possible onset of phase transition and track crystal nucleation and growth. The trends of Figure 4 are noticeably clear: first the (ε = 1.0 and σ_2 = 1.2) SW system (panel a) shows a clear phase transition occurring at approximately 3 × 10^{10} MC steps. Before the onset of crystallization, the population of fivefold sites increases at small rates and is systematically higher than the fraction of closed packed sites. At the transition point there exists a sharp increase of sites with HCP, FCC and FIV similarity. Immediately after the transition, the structure stabilizes with the fraction of close packed sites accounting for more than 50% of the total population. We should also remind again that spheres lying on the cluster surface have incomplete Voronoi polyhedra and, by definition, extremely high CCE norm (HCP or FCC). Additionally, the sharp increase in the number of crystal sites is accompanied by a significant drop of the fivefold population. In the final stable structure, fivefold sites, even in reduced numbers, co-exist with the established crystal morphology. Based on past studies [28,29] such trend points towards the self-assembly of fivefold sites into specific geometric patterns. Further confirmation will be provided in the continuation through visual inspections of the corresponding system configurations. The (ε = 2.0 and σ_2 = 1.2) SW system (panel b) exhibits different behavior: the fraction of HCP sites remains, within fluctuations, constant during the simulation while the FCC and FIV ones keep increasing slowly until they reach plateau values. In the corresponding configurations the population of fivefold sites is

systematically higher than the combined population of close packed sites. In this case there is a clear competition between fivefold local symmetry and crystal nucleation. Strong attraction, as indicated by the elevated value ε = 2.0, favors local symmetry and the formation of fivefold sites. Their population is so abundant that effectively hinders crystallization and leads to glass formation. As mentioned earlier the observed trends do not change even if we appreciably extend the duration of the MC simulation. In system (ε = 0.5 and σ_2 = 2.0), as seen in panel (c) of Figure 4 HCP population increases continuously until 10^{10} MC steps while the FIV and FCC populations remain constant. Then, a substantial growth occurs for the HCP population while both the FIV and FCC ones drop to zero. Again, the transition is sharp as it takes place within a couple of simulation frames. The final resulting configuration is of pure HCP character and it amounts for more than 50% of the total monomers.

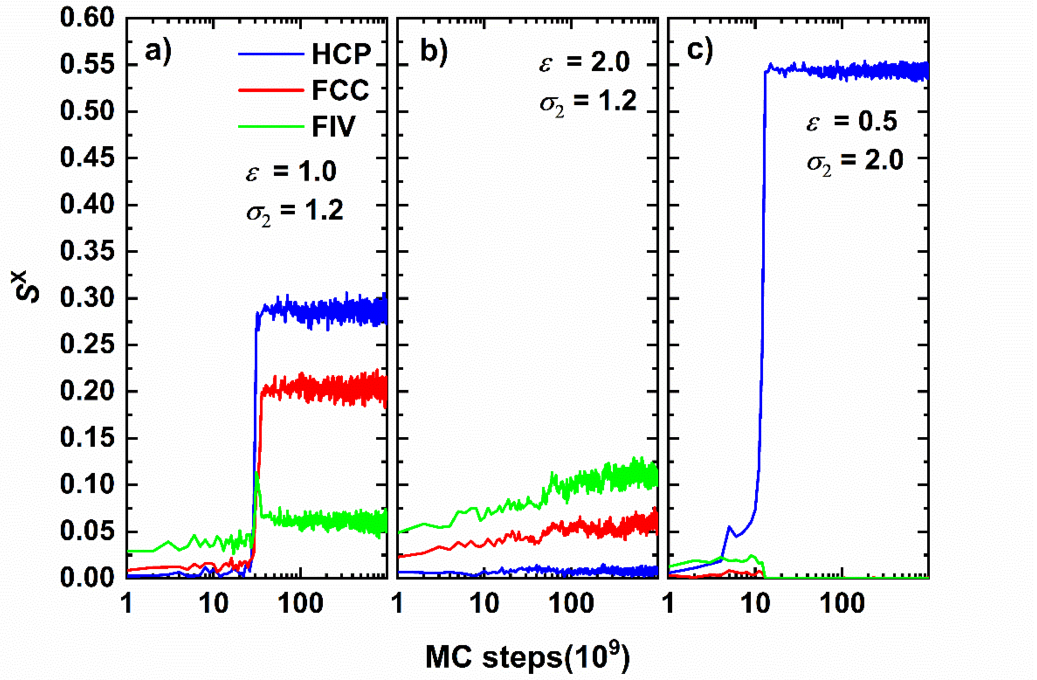

Figure 4. CCE-based order parameter, S^X, (where X is HCP, FCC or FIV) as a function of Monte Carlo steps for the SW chain systems: (**a**) $\varepsilon = 1.0$ and $\sigma_2 = 1.2$; (**b**) $\varepsilon = 2.0$ and $\sigma_2 = 1.2$; (**c**) $\varepsilon = 0.5$ and $\sigma_2 = 2.0$ in log-linear scale.

With the information of the structural order parameters, accumulated over all simulated systems, in hand we can construct the phase diagram of the SW chains. This is quantified by the dependence of the degree of crystallinity and the fivefold order parameter on the interaction parameters. Figure 5 shows τ^c (left y-axis) and S^{FIV} (right y-axis) versus ε in log-linear scale keeping the interaction range fixed at σ_2 = 1.2. Three different phase regimes exist with the corresponding onsets being recognized by the dashed vertical lines. In the regime of weak attraction ($0 \leq \varepsilon \leq 0.8$) clusters are formed but either they are unstable and do not grow further (low-ε regime) or they form a single cluster which remains amorphous with no traces of crystal or fivefold nuclei. As the first threshold is approached, we detect a small but systematic increase of fivefold symmetry: in the assembled cluster local configurations appear that favor short-range, non-crystallographic order. The second regime ($0.9 \leq \varepsilon \leq 2.0$) is characterized by a very sharp increase in the degree of crystallinity. Hence, in this intensity range the SW chain system shows phase (amorphous to crystal) transition. As suggested by the fluctuations in the number of fivefold sites, the established morphologies may correspond to fivefold-free crystals or to crystals possessing significant structural defects in the form of twins. Given the competition between fivefold and close packing in the case of zero fivefold population perfect HCP or FCC crystals, or random hexagonal close packed (rhcp) layers with a single stacking direction are expected. In opposite cases, the presence of fivefolds suggests self-

assembly into organized patterns compatible with twinning. In the final phase regime ($\varepsilon > 2.0$) the situation is drastically different: fivefold population increases significantly and surpasses the one of close packed sites. This is a result of the high intensity, the equivalent to high quench rate in the MD-based cooling of bead-spring chains in Ref. [62], which leads to a vitrified (glassy) structure lacking long-range order. From past simulation studies and from the structural point of view it has been established that crystal nuclei grow in regions far from ones populated by fivefolds [28,29]. Given that strong attraction leads to abundant fivefold symmetry in all related SW chain systems, combined with the fact that the formed cluster has specific and finite dimensions, crystal nuclei, once they are formed, do not have the necessary space to grow further. Consequently, the stable structures in this regime are characterized by increased numbers of fivefold sites and isolated crystal nuclei.

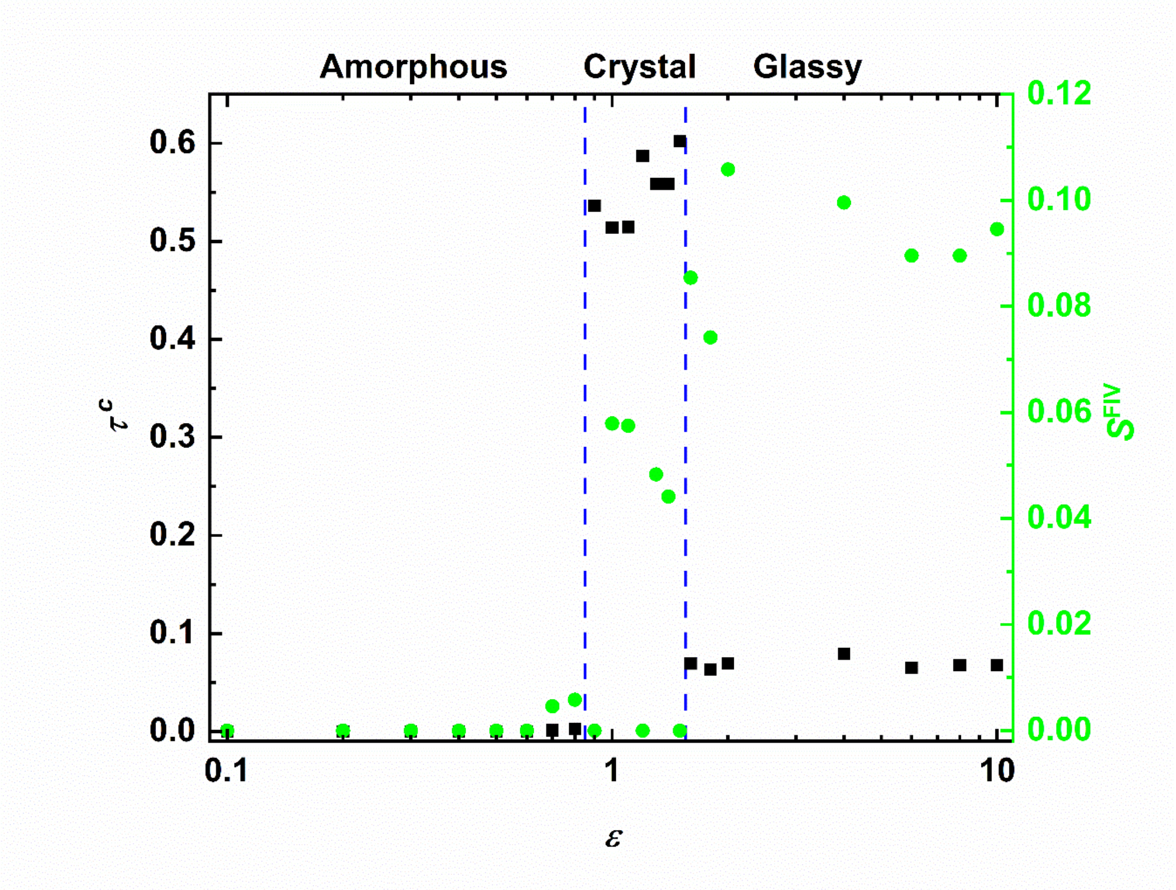

Figure 5. (**Left** y-axis) Degree of crystallinity, τ^c, (= S^{HCP} + S^{FCC}) and (**right** y-axis) fivefold order parameter, S^{FIV}, as a function of the interaction intensity, ε, of the square well potential for a fixed range (σ_2 = 1.2) in log-linear scale. Vertical dashed, blue lines indicate the transition from amorphous to crystal and from crystal to fivefold-rich glassy regimes as dictated by the population of closed packed and fivefold sites and the corresponding established morphologies.

The phase behavior of the SW chains as a function of the interaction range, keeping the intensity fixed at ε = 0.5, is presented in Figure 6. Similar trends are established with the same three distinct phase regimes appearing. First, for short range ($1 \leq \sigma_2 \leq 1.8$) there are neither close packed nor fivefold sites. Close to the phase threshold, we again observe an increase of the fivefold fraction. Given the similarity in both plots this small but non-negligible increase in fivefold symmetry can thus be considered as a hindsight for the commencement of crystallization. The second regime ($1.9 \leq \sigma_2 \leq 2.4$) is again marked by crystallization. In contrast to the intensity-based phase behavior, here in the crystal regime all simulated systems resulted in fivefold-free ordered morphologies of perfect crystals. In the final phase regime of glass formation ($\sigma_2 \geq 2.6$) the population of fivefold symmetric sites equals or exceeds that of close packed crystals. Crystallization is inhibited, and a disordered,

vitrified structure is again obtained being intimately related to the abundance of fivefold symmetry leading to structural arrest.

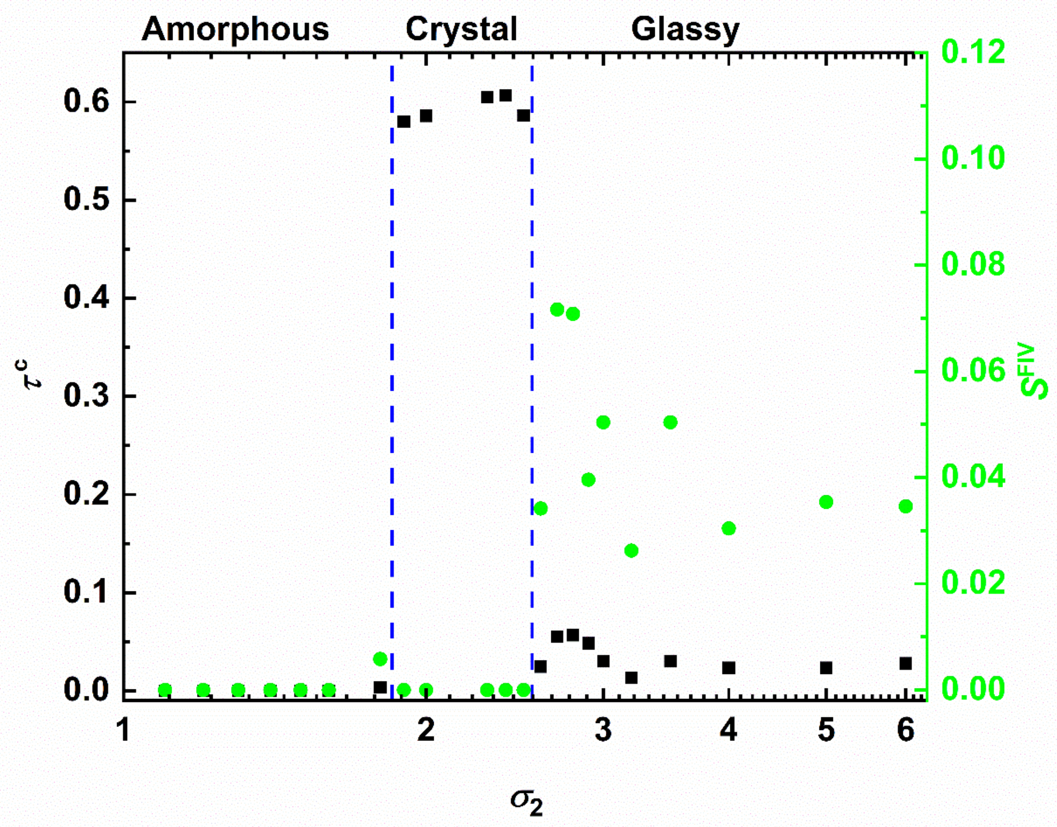

Figure 6. (**Left** *y*-axis) Degree of crystallinity, τ^c, (= S^{HCP} + S^{FCC}) and (**right** *y*-axis) fivefold order parameter, S^{FIV}, as a function of the interaction range, σ_2, of the square well potential for a fixed intensity (ε = 0.5) in log-linear scale. Vertical dashed, blue lines indicate the transition from amorphous to crystal and from crystal to fivefold-rich glassy regimes as dictated by the population of closed packed and fivefold sites and the corresponding established morphologies.

Up to this point crystallization and glass formation and the corresponding transitions have been quantified through metrics related to the CCE norm. In the continuation, after quantitative assessment, we visually inspect the amorphous, crystalline or fivefold-abundant glassy morphologies at the end of the MC simulations (Figure 7). In all snapshots, monomers are color-coded according to their CCE norm, with amorphous sites (i.e., sites with neither HCP/FCC nor FIV character) having reduced radii to improve clarity. This is because, as stated earlier, the monomers lying on the surface of the cluster have remarkably high CCE norms with respect to all reference structures and further obscure the view of the inner cluster segments. In the top row of Figure 7 from left to right snapshots are shown with increasing intensity at fixed range (σ_2 = 1.2). In the bottom row, configurations are ordered by increasing range at constant attraction strength (ε = 0.5).

Both classifications show identical trends. First, weak interaction leads to a small number of clusters, none of which shows any traces of crystals or fivefolds (panel a). Increasing attraction leads to well defined ordered morphologies. These range from crystals of mixed HCP/FCC structures with random orientation, multiple stackings with twin defects at the boundaries (panel b) to random hexagonal close packed (rhcp) alternating layers of unique stacking direction (panel c). Stronger attraction leads to suppression of crystallization and to the appearance of numerous sites with fivefold symmetry randomly dispersed in the cluster (panel d). As close packed sites are in the minority and remain isolated, further crystal growth is hindered leading to fivefold abundant

vitrified clusters. Similarly, increasing interaction range drives the system from the initially amorphous structure (panel e) to perfect HCP (panel f) and FCC (panel g) crystals and finally to fivefold-dominated glassy assemblies (panel h). It is interesting to notice that the structures obtained by tuning the attraction range correspond to perfect close packed morphologies, while the ones obtained by tuning the attraction intensity result in crystals of mixed HCP/FCC character, usually with structural defects in the form of twins.

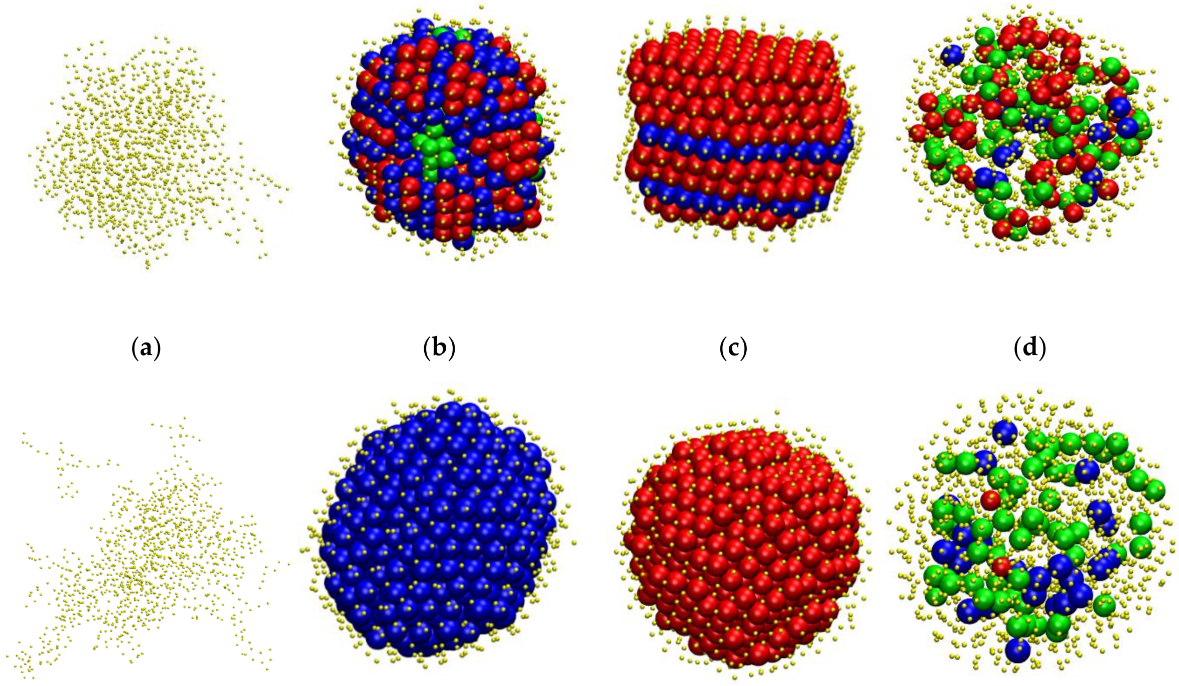

(**a**) (**b**) (**c**) (**d**)

(**e**) (**f**) (**g**) (**h**)

Figure 7. Snapshots of system configurations at the end of the MC simulation corresponding to the following sets of interaction intensity, ε, and range, σ_2. Top row: fixed range σ_2 = 1.2 and (**a**) ε = 0.6; (**b**) ε = 1.0; (**c**) ε = 1.2; (**d**): ε = 2.0. Bottom row: fixed intensity ε = 0.5 and (**e**) σ_2 = 1.2; (**f**) σ_2 = 2.0; (**g**) σ_2 = 2.5; (**h**) σ_2 = 3.0. Sphere monomers are color-coded according to the CCE norm: Blue, red and green correspond to HCP-, FCC- and FIV- like sites, respectively. Amorphous (neither HCP/FCC nor FIV) monomers are shown in yellow and with reduced radii to enhance clarity. 3-D, interactive versions of the figure panels exist in supplement material (as well as a 3-D, interactive version of the present manuscript).

Figure 8 presents in more detail selected chain morphologies containing fivefold symmetry. In the upper panels the whole system is visualized including close packed nuclei or crystals, while in the lower panels only fivefold and amorphous sites are shown. Isolating the fivefold structures allows us to understand their possible self-assembly and organization especially when co-existing with crystals. In parallel, beyond visual inspection the complete information of sphere coordinates, considering periodic boundary conditions, allows us to perform a twin element analysis based on Koch's standard International Union of Crystallography calculation [73], following the steps of Ref. [29]. Linear arrangements of fivefolds have a structure of multiple, cyclic twin. For the cyclic twin morphologies, we can observe that numerous twin sectors have been fully assembled in spite of the finite size of the encasing cluster. When twin boundaries meet, the corresponding boundaries consist of fivefold sites as imposed by the twin composition law. According to panel (ii) the axis consists of spheres which show pseudo fivefold symmetry. The structure can be viewed effectively as a linear array of parallel stacked pentagons or a pile of pentagonal bipyramids [3,74] reminiscent of concentric pentagons of the Bagley packing sequence [75].

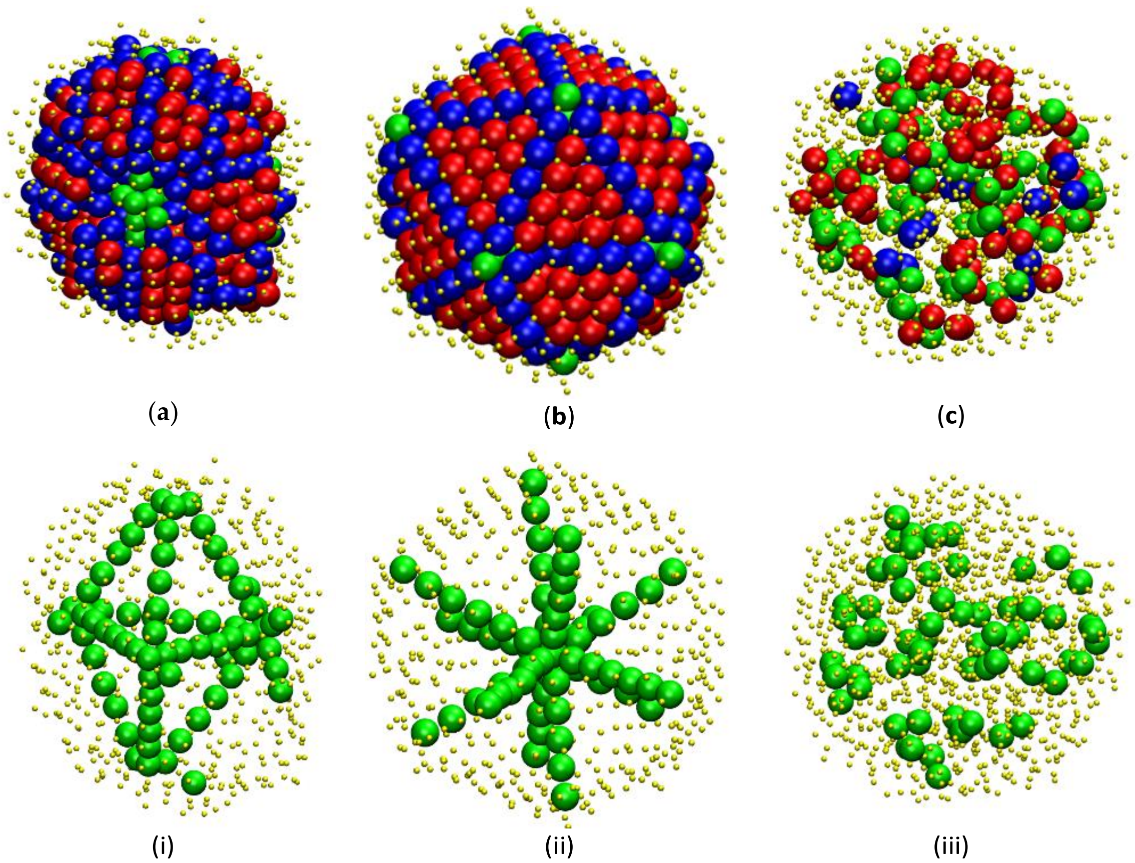

Figure 8. Top row (panels **a–d**): system snapshots of square well chains interacting with the following parameters: (**a**) ε = 1.0 and σ_2 = 1.2; (**b**) ε = 1.3 and σ_2 = 1.2; (**c**) ε = 2.0 and σ_2 = 1.2. Sphere monomers are color-coded according to the CCE norm: Blue, red and green correspond to HCP-, FCC- and FIV-like sites, respectively. Amorphous (neither HCP/FCC nor FIV) monomers are shown in yellow and with reduced radii to enhance clarity. Bottom row (panels **i–iii**): same configurations but showing only fivefold and amorphous sites. 3-D, interactive versions of the figure panels exist in supplement material (as well as a 3-D, interactive version of the present manuscript).

Based on the description above a point to notice is the similarity of these structures, whose appearance is driven by the square well interaction energy, to those of monomeric morphologies of hard spheres, driven by entropy [28,29], highlighting the universal character of fivefold symmetry in general crystallization phenomena as has been also established by MD simulations on quenched LJ chain systems [62].

As a final remark we should note that independent MC simulations have been carried out starting from different initial hard-sphere chain configurations, especially for ε and σ values near the transitions between the phase regimes (amorphous → crystal → glassy). The configurations obtained in these independent runs confirm that the thresholds and the equilibrium morphologies coincide or are similar to the ones presented here. Representative structures of such simulations are available as supplementary material.

3.3. Local Chain Structure

The phase behavior of chains as a function of attraction intensity and range has been established in the previous section. Here we analyze the short- and long-range structure of the chains as they first become segments of a cluster and successively parts of the crystal morphology (where applicable). Given that bond gap tolerance is small (dl = 6.5×10^{-4}) and bond lengths are practically constant, emphasis is placed on the distribution of bending (Figure 9) and torsion (Figure 10) angles. For comparison, the corresponding results from the hard-sphere chains (ε = 0) under the same packing density are also shown. Both distributions are obtained over all monomers of the system independently of their local structure as identified by the CCE norm. Based on this, we expect that

the sites belonging to the external surface of the cluster might, in principle, reduce the peaks that could possibly appear as a result of specific crystal morphologies or show ones that are not compatible with the observed ordered structures. As we demonstrate in the following sections, even when including the contribution of all monomers the tendencies are quite clear and easily identifiable.

We start the analysis with the bending angle, θ, which corresponds to the angle formed by successive triplets of monomers (or equivalently by two consequent unit vectors) along the chain backbone. In the legend of the graph we have marked the transitions to the three different phase regimes (amorphous ↔ crystal ↔ glass), as identified by the CCE-based results in Section 3.2. Based on the distribution curves, we can clearly distinguish three different behaviors, which follow closely the phase behavior trends. First, focusing on the effect of attraction strength (left panel of Figure 9 with fixed $\sigma_2 = 1.2$) weak interaction ($\varepsilon = 0.6$) leads to a rather uniform distribution with a small peak at 60°. This curve follows qualitatively the uniform pattern of hard-sphere chains ($\varepsilon = 0$) which is turn agrees exceptionally well with the expected distribution of isolated chains (vanishing volume fraction) computed by taking into account excluded volume interactions between atoms, i_{-1}, i and i_{+1} forming triplets (see for example Figure 1 and related discussion in the appendix of Ref. [20]). The main change with respect to the hard sphere model settles in at an angle of approximately 106.3° where a sharp increase is observed until 120° degrees. The latter is the limit value corresponding to tangency in non-overlapping triplet of hard spheres. All SW systems of the left panel of Figure 9 exhibit this increase. Its occurrence and position can be easily explained by taking into consideration that attraction range is fixed in all cases at $\sigma_2 = 1.2$. Accordingly, this is the maximum distance between spheres i_{-1} and i_{+1} to guarantee attraction. Given that, within a negligible error, bond lengths are close to unity ($dl = 6.5 \times 10^{-4}$) and by applying the cosine rule the value for the supplement of bending angle is 106.3° (for $\sigma_2 = 1.2$). Due to normalization and given that, due to attraction, for $\theta > 106.3°$ the SW distribution is significantly higher than the HS, for $\theta < 106.3°$ the SW curve should be flat and systematically below the HS one. This is verified by the data of Figure 9 (left panel). Curves corresponding to $\varepsilon = 1.0$ and 1.2 are similar, not only because the values of attraction lie close together but mainly because both systems crystallize into resembling ordered morphologies (panels b and c of Figure 7). Compared to the uniform sampling of dilute hard spheres and the small peak of the amorphous SW system, the chain crystals show prominent peaks at approximately 5, 60, 90 and 120°. Valleys are also observed mainly between 20 and 40°, around 70 and 100°. The similarity of the bending angle distribution, as obtained for SW chains forming a single energy-driven crystal cluster, and the one for entropy-driven HS chain crystals at high volume fractions (as seen in Figure 4 of Ref. [23]) is striking. The favorable local conformations are adopted due to geometric requirements as imposed by the established HCP and FCC crystal morphologies. Trying to explain the heights, if we consider the close packed crystals, the occurrence frequency of a 90° angle is calculated analytically as half that of 60° in the freely jointed model. In the final phase regime, where crystallization is inhibited, we observe significant deviations in the bending angle distribution compared to the crystal phase. The first maximum is shifted to slightly higher values while its intensity reduces. The second peak also migrates to larger bending angles (66°) but increases in strength. The most important departure comes in the vicinity of 90° where the corresponding maximum completely disappears. Another interesting point is the observed local minimum at 115° following shortly the peak at 106.3°. The combination of the absence of peak at 90° and the significant reduction of the global peak at 120° is indicative of the limited population of HCP- and FCC-like sites without resorting to the more refined CCE analysis.

Similar conclusions can be drawn by studying the effect of attraction range (right panel of Figure 9 with fixed $\varepsilon = 0.5$). The three distinct phase regimes are reflected in the distribution of bending angles. First, for the amorphous regime as range increases the observed peak, where attraction settles in, shifts to lower values. For example, from 106.3° (at $\sigma_2 = 1.2$) the maximum reduces to 73.7° (at $\sigma_2 = 1.6$) as also confirmed by simple calculations based on the cosine rule. Entering the crystal regime the curve of $\sigma_2 = 2.5$ (right panel) is similar to the curve of $\varepsilon = 1.2$ (left panel) even if one crystal is perfect FCC ($\sigma_2 = 2.5$) while the other is of mixed FCC/HCP character with twin defects ($\varepsilon = 1.2$). Again, we

should note here that in the bending angle distribution all spheres are considered, even the ones on the surface of the cluster. This explains why the results between (σ_2 = 2.0) and (σ_2 = 2.5), leading to equally perfect HCP and FCC crystals, respectively, are different.

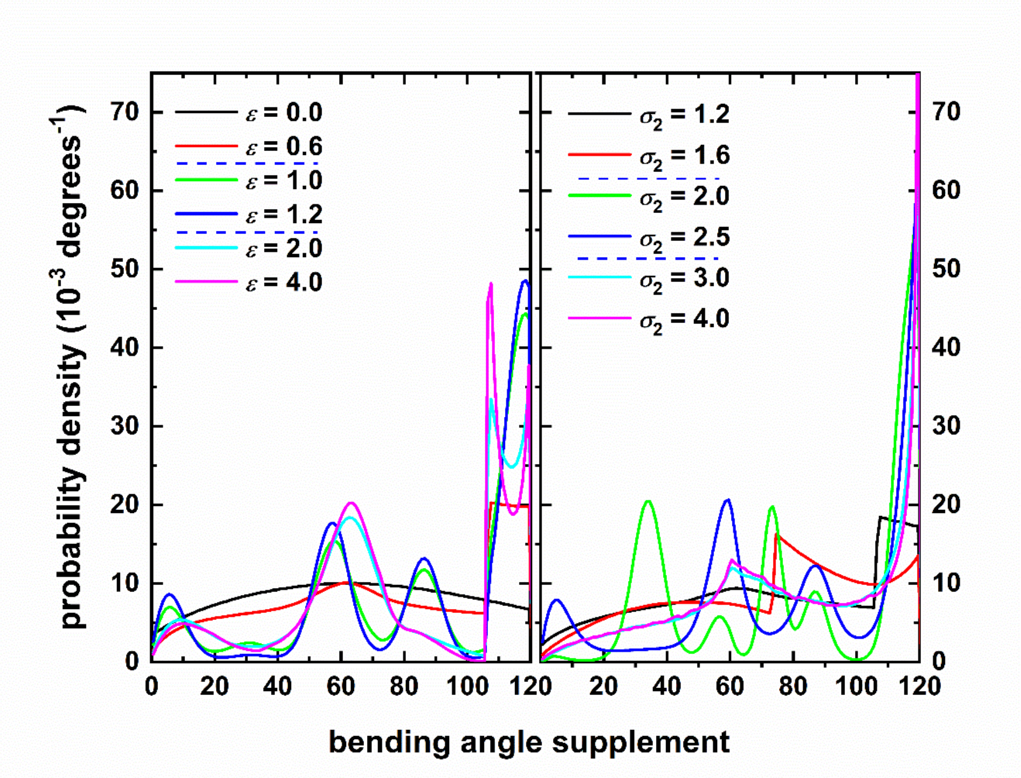

Figure 9. Distribution of bending angle supplement as a function of (**left** panel): attraction intensity for fixed range (σ_2 = 1.2) and (**right** panel): attraction range for fixed intensity (ε = 0.5). Dashed horizontal blue lines in legend identify transitions to different phase regimes: amorphous → crystalline → glassy.

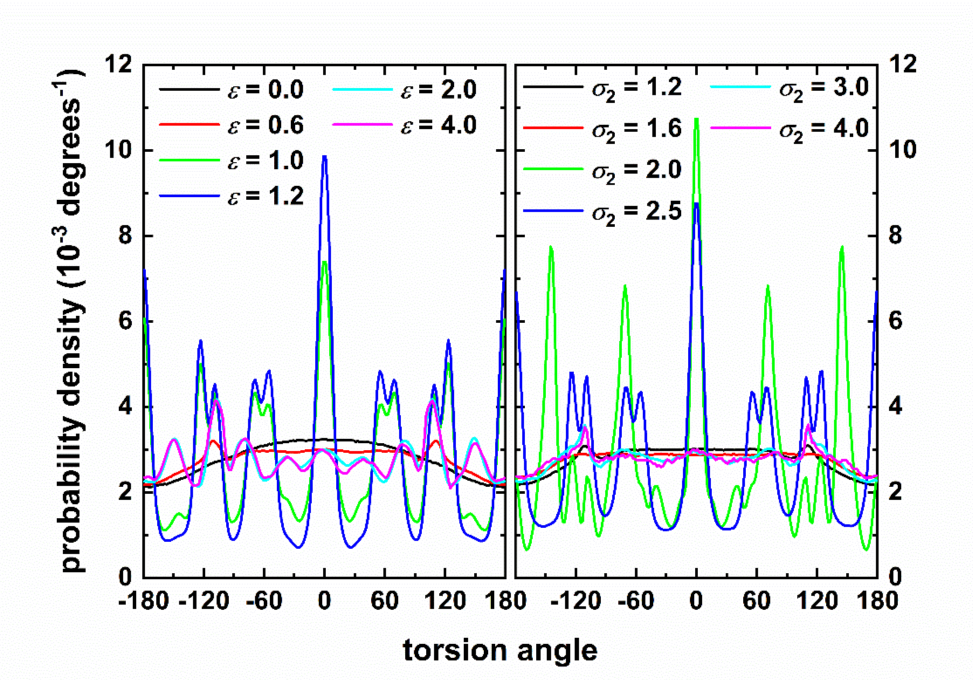

Figure 10. Torsion angle distribution as a function of (**left** panel) attraction intensity for fixed range (σ_2 = 1.2) and (**right** panel): attraction range for fixed intensity (ε = 0.5). Zero degrees correspond to trans conformation.

The torsion angle distribution as a function of intensity (left panel of Figure 10) and of range (right panel of Figure 10) confirms the findings from the analysis of the bending angle. Amorphous systems show close resemblance to the dilute HS packing. Sharp peaks are observed for all systems that crystallize, similar minima and maxima are also developed for the fivefold-rich, vitrified clusters but with intensities nowhere near those of the ordered structures. For the latter, especially prominent are the peaks at 0° (corresponding to *trans* conformation) and 180° leading to co-planar chain configurations. Peaks at 0, 54.7, 70.5, 109.5 and 180° are very reminiscent of the ones observed in dense random [20] or ordered [23] chain packings of hard spheres. Such behavior is a consequence of chains trying to adopt locally the densest possible structures and can be explained through the polytetrahedral model of disordered sphere packings [76,77]. As the system transits to the crystal state peaks at 60° and 120° tend to split into two distinct maxima; for example, the one at 111° is divided into contributions at 109.5 and 124°. Again this trend is in excellent agreement with the ones observed in the crystallization of hard-sphere chains (see for example Figure 5 of Ref. [23]).

3.4. Chain Size

Long-range chain structure is quantified here by the mean square radius of gyration, $\langle R_g^2\rangle$, and the mean square end-to-end distance, $\langle R^2\rangle$. For the freely jointed model in the limit of infinitely long chains ($N \to \infty$) it stands: $\langle R^2\rangle = 6\langle R_g^2\rangle$. Given that in the present work we model polydisperse chain systems ($N \in [6,18]$) within a single simulation we can extract the dependence on chain length. Accordingly, in the continuation angle brackets < > correspond to averaging over all chains of specific length *N* and over all recorded frames. The $\langle R_g^2\rangle$-*vs.*-*N* and $\langle R^2\rangle$-*vs.*-*N* curves are presented in the left (varied ε and fixed σ_2) and right (varied σ_2 and fixed ε) panels, respectively, of Figure 11. The first conclusion is that for all system studied $\langle R^2\rangle$ or $\langle R_g^2\rangle$ scale linearly with the number of monomers, independently of interaction type. The athermal system exhibits the largest size followed by those of weak interactions or short attraction range. The chain crystals are the ones that systematically show the shortest size especially when compared to the reference hard-sphere chain. This can be understood as monomers inside the compact cluster and/or crystal adopt configurations that minimize the local free volume, as indicated for example by the global maximum of 120° in the bending angle distribution.

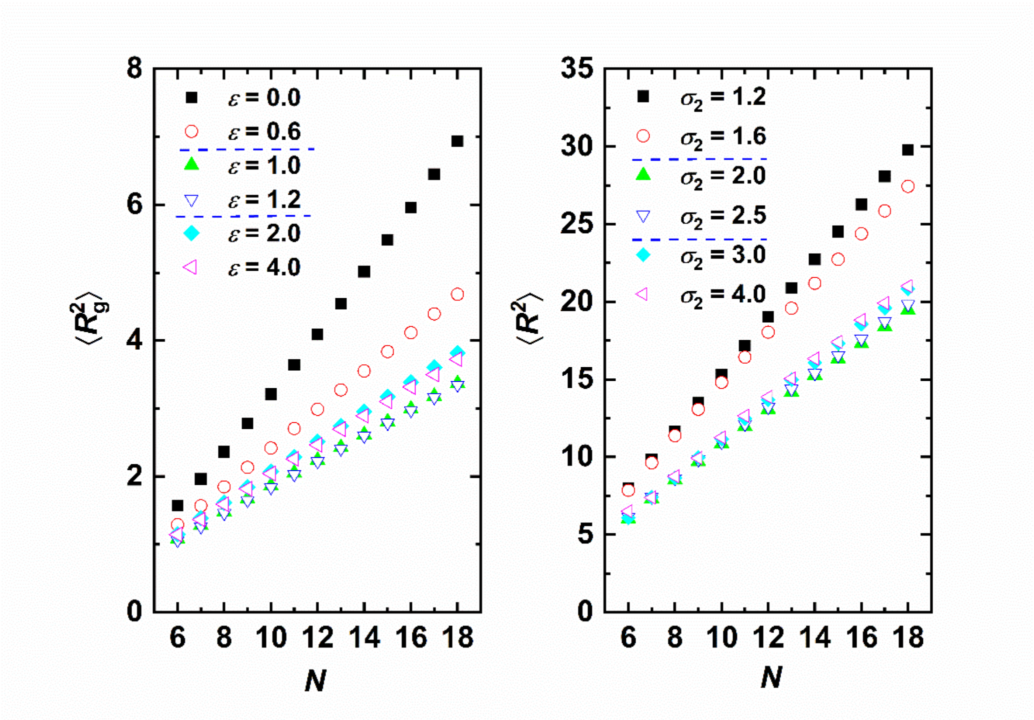

Figure 11. Effect of chain length, *N*, on chain size. **Left** panel: Mean square radius of gyration, $\langle R_g^2 \rangle$, for various attraction intensities, *ε*, with fixed range σ_2 = 1.2. **Right** panel: Mean square end-to-end distance, $\langle R^2 \rangle$, for various attraction ranges, σ_2, with fixed intensity *ε* = 0.5. Dashed horizontal blue lines in legend identify transitions to different phase regimes: amorphous → crystalline → glassy.

3.5. Local Density

To quantify cluster compactness and its correlation with morphology we calculate the local density *ρ*(*i*), around each monomer *i*, as the inverse of the volume of the Voronoi polyhedron, $V_{vor}(i)$: $\rho(i) = 1 / V_{vor}(i)$, measured in units of σ_1^{-3}. In the past, such information has provided valuable information in understanding the entropy-driven crystallization in athermal chain packings: Analysis of the Voronoi cell revealed that local environment becomes more spherical and more symmetric as the system transits to the crystal phase allowing for higher local mobility of monomers [22–24]. The maximum local density should correspond to the HCP or FCC perfect crystals (coordination number of 12) with a value approximately equal to 1.414.

Figure 12 presents the distribution of the local density for various systems corresponding to the amorphous, crystal and glassy clusters. The *ε* and *σ* pairs have been selected to match the final, stable morphologies of Figure 7. Amorphous clusters are characterized by exceptionally low density compared to crystal and glassy ones with only a very small fraction of sites having dense local environment. Crystal clusters are much more compact: the distribution becomes very narrow and concentrated at high values with the long tail corresponding to sites belonging to the external surface and having incomplete Voronoi cell. The latter corresponds to infinite volume or equivalently zero density. Between the two different crystals the "perfect" HCP one (blue line, *ε* = 0.5 and *σ* = 2.0) shows significantly denser structure and narrower distribution than the crystal of mixed character (green line, *ε* = 1.0 and *σ* = 1.2). Quotes "" are used in the sentence above to avoid confusion with the perfect HCP crystal of 12 tangent neighbors whose arrangement leads to a packing density of 0.7404 (local number density of 1.414). Here, we use the term "perfect" to identify a cluster whose sites, in their vast majority, possess a CCE norm lower than the critical threshold of similarity: $\mu < \mu^c$ (for example the crystal clusters of panels (f) and (g) in Figure 7). In strict crystallographic terms such computer-generated sites may still show structural defects compared to the perfect reference crystal as their CCE norms are very low but not zero. The vitrified (glassy) clusters also display a very high level of compactness. The distribution maxima are significantly lower than the ones of crystals and the distribution is wider while keeping the symmetric shape. It is interesting to notice that there exists an

appreciable fraction of sites with local density higher than that of the monomers belonging to crystalline clusters. This confirms past findings from hard sphere chains that the combination of symmetric arrangement and equivalence of the local environment is a key driving factor for crystallization. In both cases it is also clear that the observed morphologies are compact as a significant fraction of local densities is around 7% lower than the maximum possible for non-overlapping spheres in 3-D space.

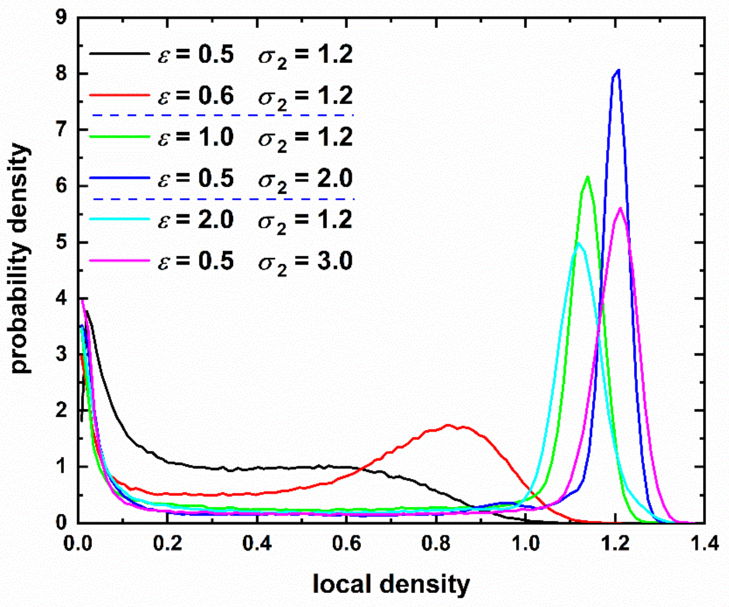

Figure 12. Distribution of local density for various pairs of intensity, ε, and range, σ, of the square well potential. Local density is calculated as the inverse of the volume of the Voronoi cell around each monomer. The corresponding final morphologies can be seen in Figure 7. Dashed horizontal blue lines in legend identify transitions to different phase regimes: amorphous → crystalline → glassy.

Figure 13 shows the dependence of the local density on the distance from the center of mass of the cluster, d_{cm}. The latter is calculated by considering all monomers having the same (unit) mass. The last 20,000 frames of each MC trajectory have been used to smoothen the curves with the local density being calculated as the average over all sites with equivalent distances from the center of mass. The conclusion that can be drawn from the data of Figure 13 is that up to a thickness of 6 layers (measured in units of the sphere diameter) the cluster remains uniformly very compact. The high local density starts to decrease only when we reach the outer layer and expectedly diminishes on the surface. The decline in density starts earlier for the imperfect crystal (of mixed HCP/FCC character), at around d_{cm} = 5. The fivefold-rich glassy cluster (ε = 0.5, σ_2 = 3.0) shows the same trends as the "perfect" HCP crystal (ε = 0.5, σ_2 = 2.0) although with significantly larger fluctuations especially close to the center. Thus, the fivefold-abundant glassy structures are as densely packed as the ordered analogs.

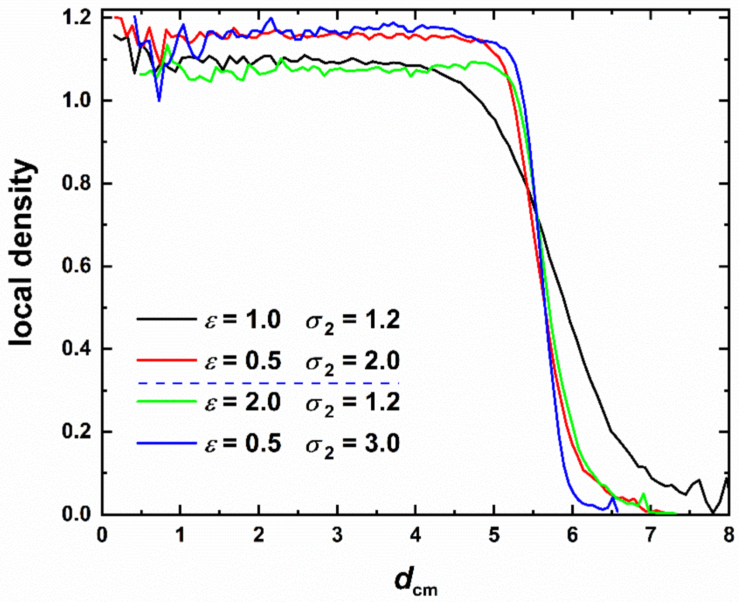

Figure 13. Local density as a function of the distance from the center of the cluster, d_{cm}, for SW chain systems corresponding to different pairs of intensity, ε, and range, σ_2, of interactions. Dashed horizontal blue line in legend identifies transition between the crystal and glass phase regimes.

The correlation between the compactness of the local environment and its similarity to a perfect crystal or fivefold local symmetry is given in Figure 14 as a parity plot between $\rho(i)$ and the minimum CCE value (i.e., min[$\mu^{HCP}(i)$, $\mu^{FCC}(i)$, $\mu^{FIV}(i)$]). For all systems including the amorphous ones, even if the average CCE norm is well above the threshold of $\mu^c = 0.245$, there is a clear negative correlation between the minimum crystallographic norm value and the corresponding density: the higher the local density the lower the corresponding CCE norm of the site.

For the crystal clusters there is a clear tendency for the ordered sites to be concentrated in the range of very high density values. A gap can be observed in the continuity near the threshold transition ($\mu^c = 0.245$), as indicated by the dashed vertical line in the panels of Figure 14. Glassy systems do not show this discontinuity and the transition before and after the crystal onset is quite smooth. Only the glass cluster corresponding to ($\varepsilon = 2.0$, $\sigma_2 = 1.2$) shows dispersion of density values as a function of the CCE-based structural similarity in the low-CCE regime of $\mu^c < 0.245$.

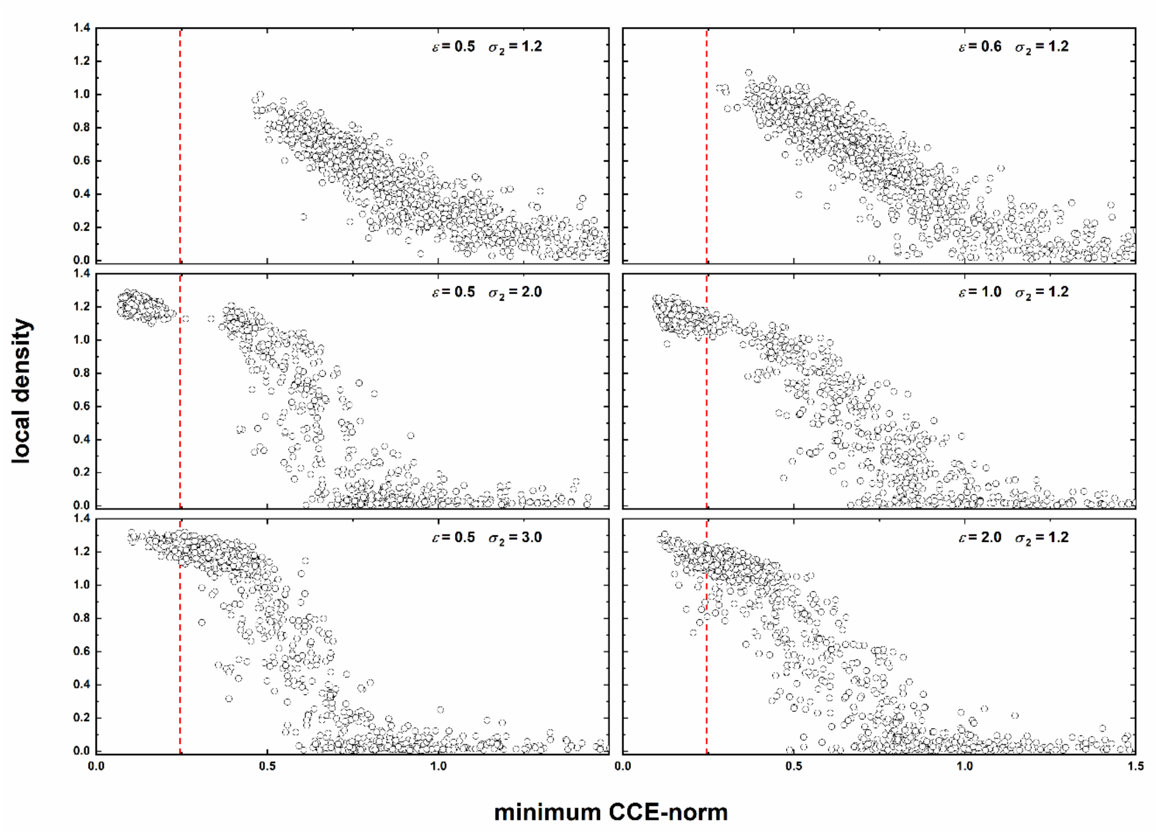

Figure 14. Parity plot of the local density as a function of the minimum CCE norm for all sites of the last recorded configuration for: (**top** panel) amorphous, (**middle** panel) crystal, and (**bottom** panel) glassy SW chain clusters. The vertical red dashed line denotes the threshold value ($\mu^c = 0.245$) for the identification of HCP, FCC or FIV local environments for non-overlapping spheres.

3.6. Pair Radial Distribution Function

Radial distribution functions provide valuable information on the structure of the cluster. The total pair radial distribution function, $g_{tot}(r)$, the intermolecular pair distribution function, $g_{inter}(r)$ and the intramolecular pair density function $w_{intra}(r)$ are shown in Figure 15. Systems have been selected to correspond to amorphous, crystalline, and glassy clusters, representative of the whole range of phase behavior. Strong peaks with regularity can be easily identified for the crystal systems (red lines) while such minima and maxima further exist in the glassy region but with reduced strength and regularity. For all systems, the initial extended plateau regime in all intra-, inter- and total distributions is related to the attraction range, σ_2, of the square well potential. The well width is responsible for the sharp drop in density immediately after $r = \sigma_2$, i.e., once the attraction range terminates, and for the "packing holes" in both intra- and inter-molecular contributions. Early peaks in w_{intra} correspond to the distance imposed by bond tangency (at $r = 1$, whose full intensity is not shown in the graph) and to configurations adopted by the bending angles as shown in Figure 9 which are absent in the amorphous case. Independently of the phase regime the structural differences in the intramolecular density disappear once we reach distances around $r = 3.5$ in agreement with past simulation findings on dense, hard-sphere chain packings [23]. With respect to the intermolecular part, as expected, distinct behaviors exist depending on the cluster being amorphous, glassy or crystalline. In the former, regularity is absent while in the latter long-range order prevails up to distances that span the whole cluster. Notwithstanding the fact that the enclosing cluster is not a perfectly symmetric sphere the observed long-range order in the g_{inter}-*vs.*-r plots provides a clear signature of the crystalline nature of the established chain morphologies. The fivefold-rich glasses show an intermediate behavior with regularity and short-range order as in the crystal counterparts but with significantly weakened intensity as radial distance increases. Apart from the maximum at $r = 1$ that corresponds to the nearest neighbors (coordination number) successive peaks at

approximately 1.41 ($\sqrt{2}$) and 1.73 ($\sqrt{3}$) correspond to second- and third-nearest neighbors, respectively, in accordance with packing structures exhibited in quenched systems of LJ chains [62].

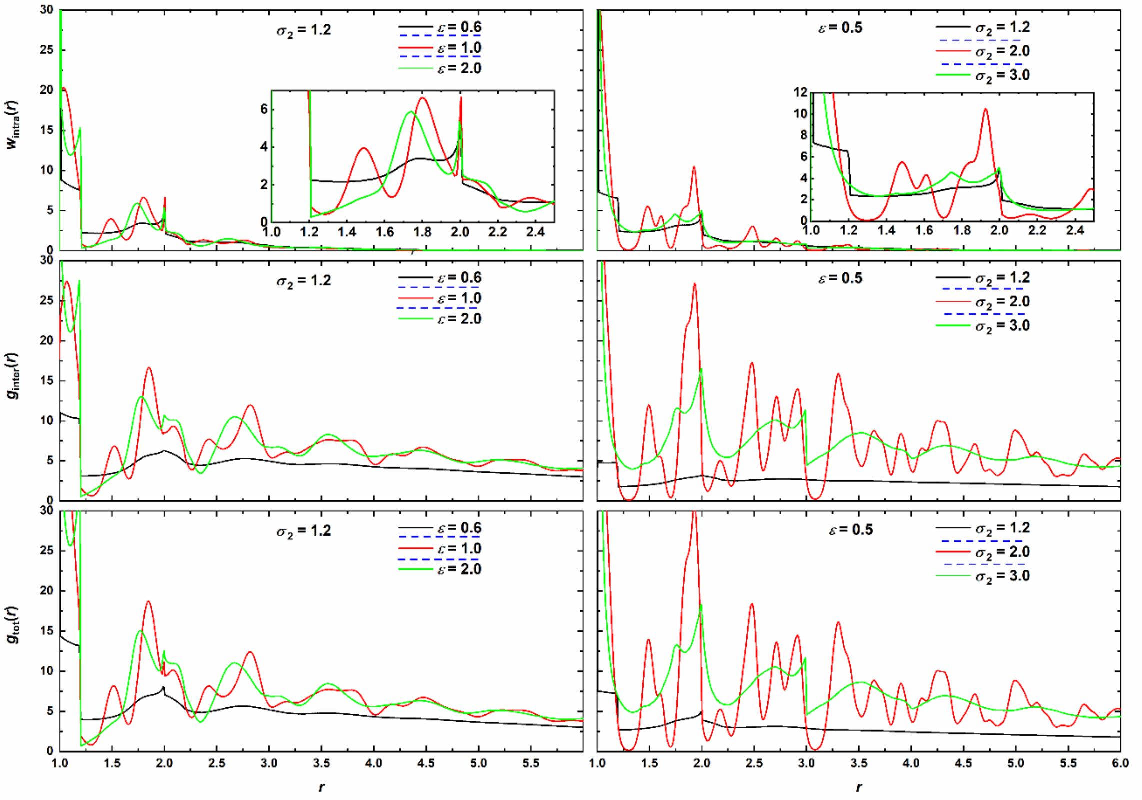

Figure 15. (**Top** panels) Intramolecular density function, $w_{intra}(r)$; (**middle** panels) intermolecular pair distribution function, $g_{inter}(r)$ and (**bottom** panels) total pair radial distribution function, $g_{tot}(r)$ as functions of radial distance, r, for (left column) various attraction intensities, ε, for fixed range (σ_2 = 1.2) and (right column) various attraction ranges, σ_2, for fixed intensity (ε = 0.5). Black, red and green correspond to SW chain clusters being amorphous, crystalline and glassy, respectively. Insets in the w_{intra} panels correspond to zoom into short radial distances and the analogous peaks. Dashed horizontal blue lines in legend identify transitions to different phase regimes: amorphous → crystalline → glassy.

4. Conclusions

In the present contribution we have studied, through Monte Carlo simulations, the phase behavior of chains whose monomers interact via the square well potential. It is found that proper tuning of the attraction intensity and range can effectively control crystal nucleation and growth in the same way as quench rates affect crystallization or glass formation in cooling processes. For a fixed range, weak attraction leads to clusters that remain amorphous with very few to no signs of crystal or fivefold morphology. Once interaction intensity passes a specific threshold crystallization is observed with a wide range of resulting ordered morphologies: from perfect close packed crystal of pure HCP or FCC character to random hexagonal close packed either with unique or with multiple stacking directions, in the latter case with defects in the form of twins. For stronger intensities, the fraction of sites with fivefold symmetry grows and exceeds that of close packed ones. Hence, crystallization is inhibited because the finite dimensions of the cluster combined with the presence of fivefold sites leads to structural arrest and vitrification thus preventing the growth of crystal nuclei. Similar phase regimes are obtained by increasing attraction range. The characteristic crystal morphologies or the fivefold-rich domains are further accompanied by monomers adopting specific configurations at the local chain level as indicated by the bending and torsion angles distribution.

Based on the above, two main conclusions can be drawn: First, the crystallization-inhibiting role of fivefold sites, as originally identified in hard-sphere packings [28,29] and more realistic quenched LJ chains [62], is further demonstrated in chain systems interacting with the square well potential. Fivefold sites are thus found to effectively suppress crystallization not only in athermal, entropy-driven packings of chains and monomers, but also in systems interacting via the square-well potential, whose crystallization is primarily energy-driven. Thus, this finding further underlines the universal character of this structural competition. Second, variation and fine tuning of the interaction parameters (intensity and range) can be used to effectively control the phase behavior of the corresponding systems. Such insights can help the design of novel colloidal and granular polymers with weak attractive interactions. Current efforts focus on studying the effect of the chain stiffness and of nanofillers on the phase behavior of polymer-based systems.

Supplementary Materials: The following are available online at www.mdpi.com/xxx/s1, Interactive, 3-D version of the panels of Figures 1, 2, 7 and 8. Additional system snapshots from independent simulations. Also available is an interactive, 3-D version of the present manuscript.

Author Contributions: Conceptualization, N.C.K. and M.L.; methodology, N.C.K., M.H. and K.F.; software (MC), M.H., M.S., N.C.K. and M.L.; software (CCE) K.F., N.C.K. and M.L.; data curation, M.H. and M.S.; writing—original draft preparation, M.H. and N.C.K.; writing—review and editing, M.S., K.F. and M.L.; visualization, M.H.; funding acquisition, K.F., N.C.K. and M.L.

Funding: This research was funded by MINECO/FEDER (Ministerio de Economia y Competitividad, Fondo Europeo de Desarrollo Regional), grant numbers "MAT2015-70478-P" and "RTI2018-097338-B-I00", and by UPM and Santander Bank, "Programa Propio UPM Santander".

Acknowledgments: Very fruitful discussions with Pablo Ramos are deeply appreciated. The authors acknowledge support through projects "MAT2015-70478-P" and "RTI2018-097338-B-I00" of MINECO/FEDER (Ministerio de Economia y Competitividad, Fondo Europeo de Desarrollo Regional). M.H. acknowledges financial support through "Program Propio UPM Santander" of UPM and Santander Bank. The authors gratefully acknowledge Universidad Politécnica de Madrid (www.upm.es) for providing computing resources on Magerit Supercomputer through projects "p208" and "r553". Authors thankfully acknowledge the computer resources at Mare Nostrum and the technical support provided by the Barcelona Supercomputing Center (BSC) through project FI-2019-2-0014.

Conflicts of Interest: The authors declare no conflict of interest.

Abbreviations

The following abbreviations are used in the manuscript:

CCE	Characteristic Crystallographic Element (norm)
FCC	Face Center Cubic
FIV	Fivefold
HCP	Hexagonal Close Packed
RHCP	Random Hexagonal Close Packed
HS	Hard Sphere
MC	Monte Carlo
MD	Molecular Dynamics
SW	Square Well
SS	Square Shoulder
LJ	Lennard-Jones

References

1. Mullin, J.W. 8 - Industrial techniques and equipment. In *Crystallization*, 4th ed.; Mullin, J.W., Ed.; Butterworth-Heinemann: Oxford, UK, 2001; pp. 315–402.
2. Reiter, G.; Sommer, J.U. *Polymer Crystallization: Obervations, Concepts and Interpretations*; Springer: Berlin/Heidelberg, Germany, 2008.

3. O'Malley, B.; Snook, I. Crystal nucleation in the hard sphere system. *Phys. Rev. Lett.* **2003**, *90*, 90, doi:10.1103/PhysRevLett.90.085702.
4. Cheng, Z.D.; Russell, W.B.; Chaikin, P.M. Controlled growth of hard-sphere colloidal crystals. *Nature* **1999**, *401*, 893–895.
5. Mullin, J.W. 6 - Crystal growth. In *Crystallization*, 4th ed.; Mullin, J.W., Ed.; Butterworth-Heinemann: Oxford, UK, 2001; pp. 216–288.
6. Kawasaki, T.; Tanaka, H. Formation of a crystal nucleus from liquid. *Proc. Natl. Acad. Sci. USA* **2010**, *107*, 14036–14041, doi:10.1073/pnas.1001040107.
7. Russo, J.; Tanaka, H. The microscopic pathway to crystallization in supercooled liquids. *Sci. Rep.* **2012**, *2*, doi:10.1038/srep00505.
8. Alder, B.J.; Wainwright, T.E. Phase Transition for A Hard Sphere System. *J. Chem. Phys.* **1957**, *27*, 1208–1209, doi:10.1063/1.1743957.
9. Frenkel, D. Entropy-driven phase transitions. *Phys. A* **1999**, *263*, 26–38, doi:10.1016/s0378-4371(98)00501-9.
10. Allen, M.P.; Tildesley, D.J. *Computer Simulation of Liquids*; Oxford University Press: New York, NY, USA, 1987.
11. Frenkel, D.; Smit, B. *Understanding Molecular Simulation: From Algorithms to Applications*, 2nd ed.; Academic Press: San Diego, CA, USA, 2002.
12. deGennes, P.G. *Scaling Concepts in Polymer Physics*; Cornell University Press: Ithaca, NY, USA, 1980.
13. Doi, M.; Edwards, S.F. *The Theory of Polymer Dynamics*; Clarendon Press: Oxford, UK, 1988.
14. Armitstead, K.; Goldbeck-Wood, G.; Keller, A. Polymer crystallization theories. *Adv. Polym. Sci.* **1992**, *100*, 219–312.
15. Karak, N. *Fundamentals of Polymers: Raw Materials to Finish Products*; Prentice Hall India: New Delhi, India, 2009.
16. Forrest, J.A.; Dalnoki-Veress, K. The glass transition in thin polymer films. *Adv. Colloid Interface Sci.* **2001**, *94*, 167–196, doi:10.1016/s0001-8686(01)00060-4.
17. Liu, Y.X.; Chen, E.Q. Polymer crystallization of ultrathin films on solid substrates. *Coord. Chem. Rev.* **2010**, *254*, 1011–1037, doi:10.1016/j.ccr.2010.02.017.
18. Karayiannis, N.C.; Laso, M. Dense and nearly jammed random packings of freely jointed chains of tangent hard spheres. *Phys. Rev. Lett.* **2008**, *100*, doi:10.1103/PhysRevLett.100.050602.
19. Karayiannis, N.C.; Foteinopoulou, K.; Laso, M. Contact network in nearly jammed disordered packings of hard-sphere chains. *Phys. Rev. E* **2009**, *80*, doi:10.1103/PhysRevE.80.011307.
20. Karayiannis, N.C.; Foteinopoulou, K.; Laso, M. The structure of random packings of freely jointed chains of tangent hard spheres. *J. Chem. Phys.* **2009**, *130*, 164908, doi:10.1063/1.3117903.
21. Laso, M.; Karayiannis, N.C.; Foteinopoulou, K.; Mansfield, M.L.; Kroger, M. Random packing of model polymers: Local structure, topological hindrance and universal scaling. *Soft Matter* **2009**, *5*, 1762–1770, doi:10.1039/b820264h.
22. Karayiannis, N.C.; Foteinopoulou, K.; Laso, M. Entropy-Driven Crystallization in Dense Systems of Athermal Chain Molecules. *Phys. Rev. Lett.* **2009**, *103*, doi:10.1103/PhysRevLett.103.045703.
23. Karayiannis, N.C.; Foteinopoulou, K.; Abrams, C.F.; Laso, M. Modeling of crystal nucleation and growth in athermal polymers: Self-assembly of layered nano-morphologies. *Soft Matter* **2010**, *6*, 2160–2173, doi:10.1039/b923369e.
24. Karayiannis, N.C.; Foteinopoulou, K.; Laso, M. Spontaneous Crystallization in Athermal Polymer Packings. *Int. J. Mol. Sci.* **2013**, *14*, 332–358, doi:10.3390/ijms14010332.
25. Karayiannis, N.C.; Foteinopoulou, K.; Laso, M. Jamming and crystallization in athermal polymer packings. *Philos. Mag.* **2013**, *93*, 4108–4131, doi:10.1080/14786435.2013.815377.
26. Karayiannis, N.C.; Foteinopoulou, K.; Laso, M. Twinning of Polymer Crystals Suppressed by Entropy. *Symmetry-Basel* **2014**, *6*, 758–780, doi:10.3390/sym6030758.
27. Karayiannis, N.C.; Foteinopoulou, K.; Laso, M. The role of bond tangency and bond gap in hard sphere crystallization of chains. *Soft Matter* **2015**, *11*, 1688–1700, doi:10.1039/c4sm02707h.
28. Karayiannis, N.C.; Malshe, R.; de Pablo, J.J.; Laso, M. Fivefold symmetry as an inhibitor to hard-sphere crystallization. *Phys. Rev. E* **2011**, *83*, doi:10.1103/PhysRevE.83.061505.
29. Karayiannis, N.C.; Malshe, R.; Kroger, M.; de Pablo, J.J.; Laso, M. Evolution of fivefold local symmetry during crystal nucleation and growth in dense hard-sphere packings. *Soft Matter* **2012**, *8*, 844–858, doi:10.1039/c1sm06540h.

30. Foteinopoulou, K.; Karayiannis, N.C.; Laso, M. Monte Carlo simulations of densely-packed athermal polymers in the bulk and under confinement. *Chem. Eng. Sci.* **2015**, *121*, 118–132, doi:10.1016/j.ces.2014.08.021.
31. Benito, J.; Karayiannis, N.C.; Laso, M. Confined Polymers as Self-Avoiding Random Walks on Restricted Lattices. *Polymers* **2018**, *10*, 1394, doi:10.3390/polym10121394.
32. Ramos, P.M.; Karayiannis, N.C.; Laso, M. Off-lattice simulation algorithms for athermal chain molecules under extreme confinement. *J. Comput. Phys.* **2018**, *375*, 918–934, doi:10.1016/j.jcp.2018.08.052.
33. Babu, S.; Gimel, J.C.; Nicolai, T. Crystallization and dynamical arrest of attractive hard spheres. *J. Chem. Phys.* **2009**, *130*, 064504, doi:10.1063/1.3074310.
34. Klotsa, D.; Jack, R.L. Predicting the self-assembly of a model colloidal crystal. *Soft Matter* **2011**, *7*, 6294–6303, doi:10.1039/c1sm05456b.
35. Takada, S.; Hayakawa, H. Rheology of dilute cohesive granular gases. *Phys. Rev. E* **2018**, *97*, 042902, doi:10.1103/PhysRevE.97.042902.
36. Sevick, E.M.; Monson, P.A. Cluster Integrals for Square-Well Particles - Application to Percolation. *J. Chem. Phys.* **1991**, *94*, 3070–3082, doi:10.1063/1.459830.
37. Campos, L.Q.C.; Silva, C.C.D.; Apolinario, S.W.S. Structural phases of colloids interacting via a flat-well potential. *Phys. Rev. E* **2012**, *86*, doi:10.1103/PhysRevE.86.051402.
38. Campos, L.Q.C.; Apolinario, S.W.S. Structural orderings of anisotropically confined colloids interacting via a quasi-square-well potential. *Phys. Rev. E* **2015**, *91*, doi:10.1103/PhysRevE.91.012305.
39. Jungmann, R.M.; Pereira, P.C.N.; Apolinario, S.W.S. 2D melting of confined colloids with a mixture of square and triangular order. *J. Phys. -Condens. Matter* **2018**, *30*, doi:10.1088/1361-648X/aae3c5.
40. Prestipino, S.; Malescio, G. Characterization of the structural collapse undergone by an unstable system of ultrasoft particles. *Phys. A-Stat. Mech. Its Appl.* **2016**, *457*, 492–505, doi:10.1016/j.physa.2016.03.116.
41. Hoy, R.S.; O'Hern, C.S. Minimal Energy Packings and Collapse of Sticky Tangent Hard-Sphere Polymers. *Phys. Rev. Lett.* **2010**, *105*, doi:10.1103/PhysRevLett.105.068001.
42. Rohrmann, R.D.; Santos, A. Equation of state of sticky-hard-sphere fluids in the chemical-potential route. *Phys. Rev. E* **2014**, *89*, doi:10.1103/PhysRevE.89.042121.
43. Pham, K.N.; Puertas, A.M.; Bergenholtz, J.; Egelhaaf, S.U.; Moussaid, A.; Pusey, P.N.; Schofield, A.B.; Cates, M.E.; Fuchs, M.; Poon, W.C.K. Multiple glassy states in a simple model system. *Science* **2002**, *296*, 104–106, doi:10.1126/science.1068238.
44. Gazzillo, D. On the impossibility of defining adhesive hard spheres as sticky limit of a hard-sphere-Yukawa potential. *J. Chem. Phys.* **2011**, *134*, 124504, doi:10.1063/1.3569113.
45. Rao, R.V.G.; Venkatesh, R. Computation of structure factors of liquid-metals by use of the sticky-hard-sphere potential. *Phys. Rev. B* **1990**, *42*, 1205–1208, doi:10.1103/PhysRevB.42.1205.
46. Munao, G.; O'Toole, P.; Hudson, T.S.; Costa, D.; Caccamo, C.; Giacometti, A.; Sciortino, F. Phase separation and self-assembly of colloidal dimers with tunable attractive strength: From symmetrical square-wells to Janus dumbbells. *Soft Matter* **2014**, *10*, 5269–5279, doi:10.1039/c4sm00544a.
47. O'Toole, P.; Munao, G.; Giacometti, A.; Hudson, T.S. Self-assembly behaviour of hetero-nuclear Janus dumbbells. *Soft Matter* **2017**, *13*, 7141–7153, doi:10.1039/c7sm01401e.
48. Kuriata, A.; Sikorski, A. Collapse Transition of Cyclic Homopolymers and Block Copolymers. *Macromol. Theory Simul.* **2018**, *27*, doi:10.1002/mats.201700089.
49. Zierenberg, J.; Marenz, M.; Janke, W. Dilute Semiflexible Polymers with Attraction: Collapse, Folding and Aggregation. *Polymers* **2016**, *8*, 333, doi:10.3390/polym8090333.
50. Taylor, M.P.; Aung, P.P.; Paul, W. Partition function zeros and phase transitions for a square-well polymer chain. *Phys. Rev. E* **2013**, *88*, doi:10.1103/physreve.88.012604.
51. Taylor, M.P.; Paul, W.; Binder, K. Phase transitions of a single polymer chain: A Wang–Landau simulation study. *J. Chem. Phys.* **2009**, *131*, 114907, doi:10.1063/1.3227751.
52. Zhou, Y.; Hall, C.K.; Karplus, M. First-Order Disorder-to-Order Transition in an Isolated Homopolymer Model. *Phys. Rev. Lett.* **1996**, *77*, 2822–2825, doi:10.1103/physrevlett.77.2822.
53. Zhou, Y.; Karplus, M.; Wichert, J.M.; Hall, C.K. Equilibrium thermodynamics of homopolymers and clusters: Molecular dynamics and Monte Carlo simulations of systems with square-well interactions. *J. Chem. Phys.* **1997**, *107*, 10691–10708, doi:10.1063/1.474186.
54. Schnabel, S.; Vogel, T.; Bachmann, M.; Janke, W. Surface effects in the crystallization process of elastic flexible polymers. *Chem. Phys. Lett.* **2009**, *476*, 201–204, doi:10.1016/j.cplett.2009.05.052.

55. Schnabel, S.; Bachmann, M.; Janke, W. Elastic Lennard-Jones polymers meet clusters: Differences and similarities. *J. Chem. Phys.* **2009**, *131*, doi:10.1063/1.3223720.
56. Schnabel, S.; Janke, W.; Bachmann, M. Advanced multicanonical Monte Carlo methods for efficient simulations of nucleation processes of polymers. *J. Comput. Phys.* **2011**, *230*, 4454–4465, doi:10.1016/j.jcp.2011.02.018.
57. Gross, J.; Neuhaus, T.; Vogel, T.; Bachmann, M. Effects of the interaction range on structural phases of flexible polymers. *J. Chem. Phys.* **2013**, *138*, 074905, doi:10.1063/1.4790615.
58. Braier, P.A.; Berry, R.S.; Wales, D.J. How the range of pair interactions governs features of multidimensional potentials. *J. Chem. Phys.* **1990**, *93*, 8745–8756, doi:10.1063/1.459263.
59. Wales, D.J. Structural and topological consequences of anisotropic interactions in clusters. *J. Chem. Soc. Faraday Trans.* **1990**, *86*, 3505–3517, doi:10.1039/FT9908603505.
60. Wales, D.J.; Berry, R.S. Melting and freezing of small argon clusters. *J. Chem. Phys.* **1990**, *92*, 4283–4295, doi:10.1063/1.457788.
61. Prabhu, A.; Babu, S.B.; Dolado, J.S.; Gimel, J.C. Brownian cluster dynamics with short range patchy interactions: Its application to polymers and step-growth polymerization. *J. Chem. Phys.* **2014**, *141*, 16, doi:10.1063/1.4886585.
62. Hoy, R.S.; Karayiannis, N.C. Simple model for chain packing and crystallization of soft colloidal polymers. *Phys. Rev. E* **2013**, *88*, doi:10.1103/PhysRevE.88.012601.
63. Nguyen, H.T.; Smith, T.B.; Hoy, R.S.; Karayiannis, N.C. Effect of chain stiffness on the competition between crystallization and glass-formation in model unentangled polymers. *J. Chem. Phys.* **2015**, *143*, 144901, doi:10.1063/1.4932193.
64. Ni, R.; Dijkstra, M. Effect of bond length fluctuations on crystal nucleation of hard bead chains. *Soft Matter* **2013**, *9*, 365–369, doi:10.1039/c2sm26969d.
65. Hino, T.; Prausnitz, J.M. A perturbed hard-sphere-chain equation of state for normal fluids and polymers using the square-well potential of variable width. *Fluid Phase Equilib.* **1997**, *138*, 105–130, doi:10.1016/s0378-3812(97)00154-4.
66. Karayiannis, N.C.; Giannousaki, A.E.; Mavrantzas, V.G.; Theodorou, D.N. Atomistic Monte Carlo simulation of strictly monodisperse long polyethylene melts through a generalized chain bridging algorithm. *J. Chem. Phys.* **2002**, *117*, 5465–5479, doi:10.1063/1.1499480.
67. Karayiannis, N.C.; Mavrantzas, V.G.; Theodorou, D.N. A novel Monte Carlo scheme for the rapid equilibration of atomistic model polymer systems of precisely defined molecular architecture. *Phys. Rev. Lett.* **2002**, *88*, doi:10.1103/PhysRevLett.88.105503.
68. Karayiannis, N.C.; Laso, M. Monte Carlo scheme for generation and relaxation of dense and nearly jammed random structures of freely jointed hard-sphere chains. *Macromolecules* **2008**, *41*, 1537–1551, doi:10.1021/ma702264u.
69. Hoy, R.S.; O'Hern, C.S. Glassy dynamics of crystallite formation: The role of covalent bonds. *Soft Matter* **2012**, *8*, 1215–1225, doi:10.1039/c1sm05741c.
70. Karayiannis, N.C.; Foteinopoulou, K.; Laso, M. The characteristic crystallographic element norm: A descriptor of local structure in atomistic and particulate systems. *J. Chem. Phys.* **2009**, *130*, 74704, doi:10.1063/1.3077294.
71. Giacovazzo, C.; Monaco, H.L.; Artioli, G.; Viterbo, D.; Ferraris, G.; Gilli, G.; Zanotti, G.; Gatti, M. *Fundamentals of Crystallography*; Oxford Science: Oxford, UK, 2005.
72. Humphrey, W.; Dalke, A.; Schulten, K. VMD: Visual molecular dynamics. *J. Mol. Graph. Model.* **1996**, *14*, 33–38, doi:10.1016/0263-7855(96)00018-5.
73. Koch, E. *International Tables for Crystallography, Volume C: Mathematical, Physical and Chemical Tables*, 3rd ed.; Prince, E., Ed.; Springer: New York, NY, USA, 2006.
74. Anikeenko, A.V.; Medvedev, N.N.; Bezrukov, A.; Stoyan, D. Observation of fivefold symmetry structures in computer models of dense packing of hard spheres. *J. Non-Cryst. Solids* **2007**, *353*, 3545–3549, doi:10.1016/j.jnoncrysol.2007.05.113.
75. Bagley, B.G. 5-Fold Pseudosymmetry. *Nature* **1970**, *225*, 1040–1041, doi:10.1038/2251040a0.
76. Anikeenko, A.V.; Medvedev, N.N. Polytetrahedral nature of the dense disordered packings of hard spheres. *Phys. Rev. Lett.* **2007**, *98*, 235504, doi:10.1103/PhysRevLett.98.235504.
77. Anikeenko, A.V.; Medvedev, N.N.; Aste, T. Structural and entropic insights into the nature of the random-close-packing limit. *Phys. Rev. E* **2008**, *77*, 031101, doi:10.1103/PhysRevE.77.031101.

© 2020 by the authors. Submitted for possible open access publication under the terms and conditions of the Creative Commons Attribution (CC BY) license (http://creativecommons.org/licenses/by/4.0/).
