# Supplementary material for: Crystal, Fivefold and Glass Formation in Clusters of Polymers Interacting with the Square Well Potential
Source: Polymers (Basel). 2020 May 13;12(5):1111. doi: 10.3390/polym12051111 (PMC7285265; doi:10.3390/polym12051111)
Supplement: Supplementary file 1 [file polymers-12-01111-s001.zip › supplementary_images.docx]

| 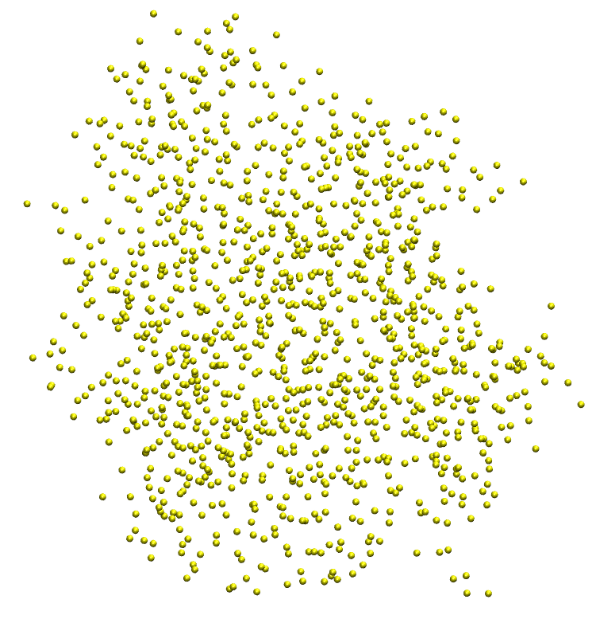  a) | 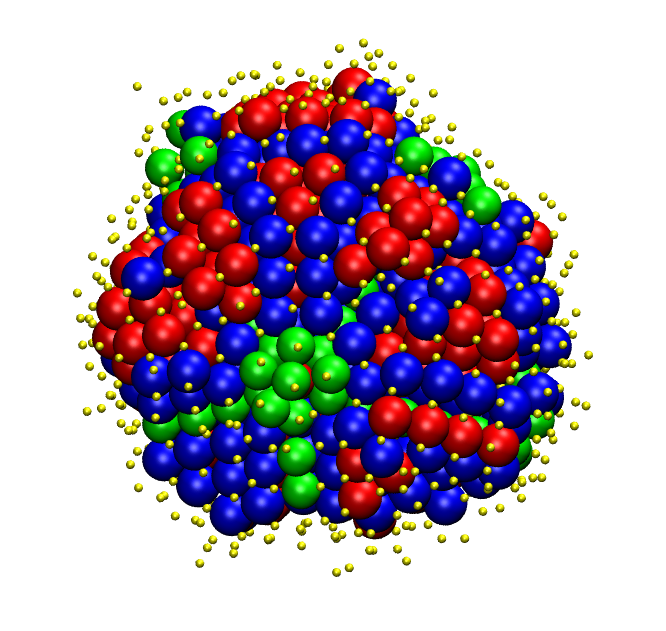  b) | 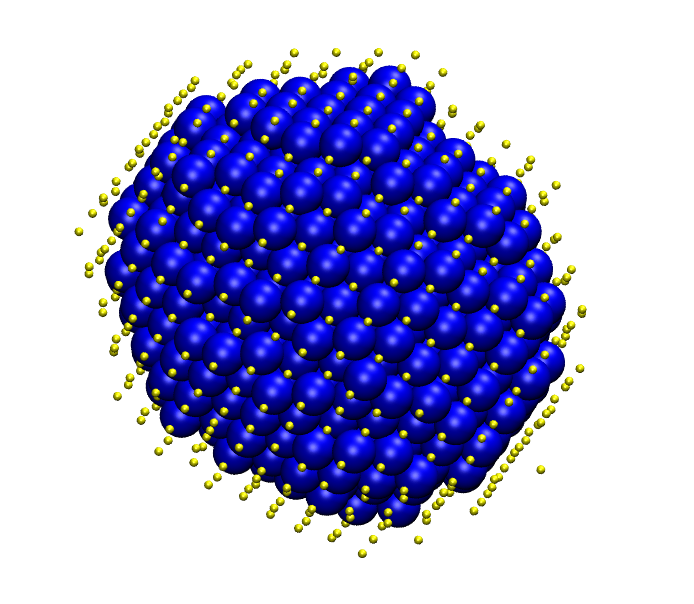  c) |
| --- | --- | --- |

**Figure Supplement Material.** Independent simulations starting from different hard-sphere chain configurations. Snapshots of system configurations at the end of the MC simulation corresponding to the following sets of interaction intensity, ε, and range, σ_2_: (a) ε = 0.5 and σ_2_ = 1.2; (b) ε = 1.0 and σ_2_ = 1.2; (c) ε = 0.5 and σ_2_ = 2.0. Sphere monomers are color-coded according to the CCE norm: Blue, red and green correspond to HCP-, FCC- and FIV- like sites, respectively. Amorphous (neither HCP/FCC nor FIV) monomers are shown in yellow and with reduced radii to enhance clarity.
